# Unravelling inclusion body myositis using a patient‐derived fibroblast model

**DOI:** 10.1002/jcsm.13178

**Published:** 2023-03-01

**Authors:** Judith Cantó‐Santos, Laura Valls‐Roca, Ester Tobías, Francesc Josep García‐García, Mariona Guitart‐Mampel, Anna Esteve‐Codina, Beatriz Martín‐Mur, Mercedes Casado, Rafael Artuch, Estel Solsona‐Vilarrasa, José Carlos Fernandez‐Checa, Carmen García‐Ruiz, Carles Rentero, Carlos Enrich, Pedro J. Moreno‐Lozano, José César Milisenda, Francesc Cardellach, Josep M. Grau‐Junyent, Glòria Garrabou

**Affiliations:** ^1^ Muscle Research and Mitochondrial Function Lab, Centre de Recerca Biomèdica CELLEX ‐ Institut d'Investigacions Biomèdiques August Pi i Sunyer (IDIBAPS) and Faculty of Medicine and Health Sciences, University of Barcelona Barcelona Spain; ^2^ Department of Internal Medicine Hospital Clinic of Barcelona Barcelona Spain; ^3^ CIBERER—Spanish Biomedical Research Centre in Rare Diseases Madrid Spain; ^4^ CNAG‐CRG, Centre for Genomic Regulation Barcelona Institute of Science and Technology Barcelona Spain; ^5^ Universitat Pompeu Fabra (UPF) Barcelona Spain; ^6^ Department of Clinical Biochemistry Institut de Recerca Sant Joan de Déu; Esplugues de Llobregat Barcelona Spain; ^7^ Department of Cell Death and Proliferation Institute of Biomedical Research of Barcelona (IIBB‐CSIC), Liver Unit‐HCB‐IDIBAPS Barcelona Spain; ^8^ CIBEREHD‐Spanish Biomedical Research Centre in Hepatic and Digestive Diseases Madrid Spain; ^9^ Department of Biomedicine, Cell Biology Unit, CELLEX‐IDIBAPS, Faculty of Medicine and Health Sciences University of Barcelona Barcelona Spain

**Keywords:** Inclusion body myositis, Myopathy, Fibroblasts, Autophagy, Inflammation, Mitochondria

## Abstract

**Background:**

Inclusion body myositis (IBM) is an inflammatory myopathy clinically characterized by proximal and distal muscle weakness, with inflammatory infiltrates, rimmed vacuoles and mitochondrial changes in muscle histopathology. There is scarce knowledge on IBM aetiology, and non‐established biomarkers or effective treatments are available, partly due to the lack of validated disease models.

**Methods:**

We have performed transcriptomics and functional validation of IBM muscle pathological hallmarks in fibroblasts from IBM patients (*n* = 14) and healthy controls (*n* = 12), paired by age and sex. The results comprise an mRNA‐seq, together with functional inflammatory, autophagy, mitochondrial and metabolic changes between patients and controls.

**Results:**

Gene expression profile of IBM vs control fibroblasts revealed 778 differentially expressed genes (*P*‐value adj < 0.05) related to inflammation, mitochondria, cell cycle regulation and metabolism. Functionally, an increased inflammatory profile was observed in IBM fibroblasts with higher supernatant cytokine secretion (three‐fold increase). Autophagy was reduced considering basal protein mediators (18.4% reduced), time‐course autophagosome formation (LC3BII 39% reduced, *P*‐value < 0.05), and autophagosome microscopic evaluation. Mitochondria displayed reduced genetic content (by 33.9%, *P*‐value < 0.05) and function (30.2%‐decrease in respiration, 45.6%‐decline in enzymatic activity (*P*‐value < 0.001), 14.3%‐higher oxidative stress, 135.2%‐increased antioxidant defence (*P*‐value < 0.05), 11.6%‐reduced mitochondrial membrane potential (*P*‐value < 0.05) and 42.8%‐reduced mitochondrial elongation (*P*‐value < 0.05)). In accordance, at the metabolite level, organic acid showed a 1.8‐fold change increase, with conserved amino acid profile. Correlating to disease evolution, oxidative stress and inflammation emerge as potential markers of prognosis.

**Conclusions:**

These findings confirm the presence of molecular disturbances in peripheral tissues from IBM patients and prompt patients' derived fibroblasts as a promising disease model, which may eventually be exported to other neuromuscular disorders. We additionally identify new molecular players in IBM associated with disease progression, setting the path to deepen in disease aetiology, in the identification of novel biomarkers or in the standardization of biomimetic platforms to assay new therapeutic strategies for preclinical studies.

## Introduction

Inclusion body myositis (IBM) is an inflammatory myopathy characterized by proximal and distal muscle weakness, inflammation, rimmed vacuoles and mitochondrial alterations in myofibres.^1^ This rare disease (OMIM 147421) is the most common acquired muscle disease in patients over 50 years old.^2^ Its prevalence ranges from 46 to 84 patients per million,^3^, ^4^ raising due to the aging of the population. The time from symptoms onset to diagnosis is delayed (around 4–5 years) with a high level of misdiagnosis, due to a lack of specialized diagnostic teams and non‐invasive biomarkers. Despite the progression towards disability being slow, effective treatments are absent, leading to an unmet medical need.^1^

Scarce knowledge has been gathered about IBM aetiology. First characterized in 1978, it is currently categorized as an inflammatory myopathy (or myositis), together with polymyositis, dermatomyositis, immune‐mediated necrotizing myopathy and non‐specific myositis.^1^ However, IBM was considered the sporadic form (sIBM) of inherited vacuolar myopathies (hIBM, caused by mutations in around 12 genes, mainly *VCP*), because both shared the presence of rimmed vacuoles. Inflammation is not present in vacuolar myopathies,^5^, ^6^ whereas it is prominent in IBM. This prompted the classification of IBM among inflammatory myopathies^2^, ^5^ and, currently, sIBM nomenclature is being moved to IBM.

The first symptoms appear in quadriceps and finger flexor muscles, leading to difficulties for climbing stairs and handling objects. In 60% of the cases, there is dysphagia and swallowing dysfunctions. The diagnosis is based on clinical and muscle histology studies, where inflammatory infiltrate, rimmed vacuoles and mitochondrial abnormalities must be present. Briefly, muscle biopsies show: (i) inflammation (CD8+ T cells, aberrant expression of MHC1 and endomysial mononuclear cell infiltrates in myofibres); (ii) degeneration (rimmed vacuoles, ubiquitin‐positive inclusions and amyloid deposits) and (iii) mitochondrial abnormalities (cytochrome c oxidase negative fibres (COX‐), succinate dehydrogenase positive cells (SDH+) and ragged‐red fibres (RRF), corresponding to an increased number of abnormal mitochondria around myofibres).^1^, ^2^, ^6^

Currently, there is no treatment for IBM. Although being an inflammatory myopathy, corticosteroids have failed, and all trials with immunosuppressants, immunomodulating agents, TGFβ inhibitors and muscle growth factors have been ineffective. Intravenous immunoglobulin treatment has been useful to treat dysphagia, despite this, IBM patients are limited to physical therapy (with difficulties for some patients) and participation in clinical trials.^7^

Validated disease models for IBM could help to gain insight into the pathophysiology of this condition, improve diagnosis and test effective treatments. However, previous attempts to develop animal models (*MCK‐βAPP* transgenic mice,^8^ *VCP* mutant mice models,^9^ and a cholesterol rabbit model^10^) revealed serious limitations, probably because they were mainly based on gene editing, and IBM is an acquired disease with poor (or merely unknown) genetic base. Consequently, most of the advances in IBM have been accomplished directly with samples from affected patients.^5^ Previous studies from our group revealed some pathological muscle features in IBM lymphocytes^11^ or alternative peripheral tissues,^12^ suggesting that IBM hallmarks could be present besides the target tissue (muscle). Considering fibroblasts are proliferative cells, used to diagnose mitochondrial diseases and to model neurodegenerative diseases,^13^, ^14^, ^15^ we tested their usefulness as a potential model to study IBM. Fibroblasts preserve genetic, epigenetic and environmental cues of affected patients, offering a low‐cost maintaining platform to deepen in disease aetiology, discover novel biomarkers and assay new therapeutic strategies for preclinical studies.

In this article, we aim to report a detailed phenotyping of fibroblasts from IBM patients compared with age and sex‐paired healthy controls (CTL), as a promising model for this disease. We offer the transcriptomic and functional assessment of this cell model together with the clinical and pathological data of included patients. Briefly, fibroblasts from IBM patients showed a clear deregulated transcriptomic pattern of 778 differentially expressed genes (DEGs) and a pathological inflammatory, degenerative and mitochondrial profile, resembling the target tissue of the disease. Additionally, the present findings prompt distinct molecular targets as relevant key players in IBM.

## Methods

### Study design and population

A case–control study was conducted in the Department of Internal Medicine from Hospital Clínic of Barcelona (Barcelona, Spain), including 14 patients and 12 healthy volunteers. IBM patients and healthy volunteers signed the informed consent (ethical code HCB/2015/0562). Additional information is provided in the Supporting Information.

### Fibroblast culture

Fibroblasts were obtained from a skin biopsy from cases and controls. Cells were harvested and collected at 80% of confluence, and phenotyped at passages 3 to 10. Briefly, fibroblasts from both groups displayed similar morphology and size. Cell growth rate was slightly reduced in IBM fibroblasts (0.019 ± 0.002 vs. 0.024 ± 0.006), although non‐significantly. Additional information is provided in the Supporting Information.

### RNA extraction, mRNA library preparation and sequencing

Total RNA was isolated from cell lysates of three IBM and three CTL fibroblasts. Differential expression analysis was performed with DESeq2 v1.26.0 R package, and genes were considered differentially expressed with an adjusted *P*‐value < 0.05 and absolute fold change |FC| > 1.5. A detailed description of mRNA libraries, sequencing and analysis is presented in the Supporting Information.

### Inflammation: Bio‐Plex assay of secreted inflammatory cytokines

A Bio‐plex assay (Bio‐Plex Pro™ Human Cytokine 27‐plex Assay #M500KCAF0Y) from Bio‐Rad (Hercules, CA, USA) was performed to quantify 27 inflammatory cytokines secreted in the supernatant of IBM and CTL fibroblasts after 96 h in culture (undiluted samples) normalized by cell number.

### Autophagy protein array

Cell lysates were obtained from IBM and CTL fibroblasts; 250–500 μg of protein per sample were loaded in the Ray Bio® C Series Human Autophagy Array 1 (Cat#: AAHATG‐1‐8, Ray Biotech, Inc., Atlanta, GA, USA), incubated overnight at 4°C and detected by chemiluminescence. Additional information is provided in Supporting Information.

### Autophagy time‐course

Autophagy flux in IBM versus CTL fibroblasts was measured in a time‐course in basal conditions (0 h), and 4 h and 8 h after the addition of 0.1 μM bafilomycin A1 from *Streptomyces griseus* (Sigma‐Aldrich® #B1793 SIGMA, Missouri, USA), and analysed through western blot analysis. Blots were probed against the anti‐ SQSTM1/p62 (Abcam #ab56416, Cambridge, UK) and anti‐LC3B (Cell Signaling #2775S, Massachusetts, USA) antibodies. More information provided in the Supporting Information.

### Immunocytochemistry for autophagosome characterization

Cells were seeded in a 16‐well glass slide (Nunc™ #178599 Lab‐Tek® Chamber Slide™, Austin, USA) at 37°C with 5% CO_2_ for 24 h. Autophagosomes were stained with anti‐LC3 pAB (MBL International® #PM036, Massachusetts, USA) and images were taken with a Zeiss LSM confocal microscope (63×). A detailed protocol is presented in the Supporting Information.

### Mitochondrial respiration

Mitochondrial respiration was measured according to the oxygen consumption of the mitochondrial respiratory chain (MRC). The oxygen consumption rate was detected with the XF Cell Mito Stress Test™ (Seahorse‐XF^e^24‐Analyser, Agilent Technologies), according to the manufacturer's protocol. Additional information is found in the Supporting Information.

### MRC enzymatic activities: COX and citrate synthase (CS) activities

COX and CS enzymatic activities were assessed in 40 μg of total protein from each fibroblast sample according to standardized protocols,^15^ at 37°C, and expressed as COX/CS ratio.

### Oxidative stress

Oxidative stress was measured through lipid peroxidation (measuring malondialdehyde (MDA) and 4‐hydroxyalkenal (HAE)) and total antioxidant capacity of the cell (TAC).^11^ Reagents and protocols are provided in the Supporting Information.

### Mitochondrial membrane potential

Mitochondrial membrane potential (MMP) was quantified with tetramethyl rhodamine methyl ester (TMRM) and detected with confocal microscopy, as the number of cells with active mitochondria per total number of cells.

### mtDNA genome content

mtDNA content was evaluated with the fragments of the mitochondrially encoded 12S rRNA gene and the nuclear‐encoded RNase‐P gene, amplified by RT‐PCR (Applied Biosystems). The relative mtDNA content was expressed as the ratio mtDNA 12S rRNA:nDNA RNase‐P.

### Mitochondrial elongation

Mitochondrial network was immunostained with TOMM20 (St Cruz Bio #sc‐11415 (1:100)), imaged with a Zeiss confocal microscope (63×) and analysed with ImageJ software using a mitochondrial morphology macro to quantify mitochondrial elongation (also called aspect ratio: AR = major axis/minor axis, where a higher AR is linked to healthier mitochondria.^16^

### Transmission electron microscopy (TEM)

Fibroblasts were fixed and incubated with 1% OsO_4_ before being sectioned using Leica ultramicrotome (Leica Microsystems). Ultrathin sections (50–70 nm) were stained and observed using a TEM, JEOL JEM‐1010 fitted with a Gatan Orius SC1000 (model 832) digital camera^17^ to seek for abnormal organelle structures. Detailed information is presented in the Supporting Information.

### Metabolite quantification

Organic acids were extracted in fibroblasts as previously reported.^18^ The trimethylsilyl derivatives obtained were separated by gas chromatography (Agilent 7890A, Wilmington, DE, USA) and detected in a mass spectrometer (Agilent 5975C, Wilmington, DE, USA). Amino acids were quantified in fibroblasts by ultra‐performance liquid chromatography coupled to tandem mass spectrometry, as previously reported.^19^ Both measurements were normalized by protein content. Additional information is detailed in the Supporting Information.

### Statistical analysis

Statistical analysis was performed with the Statistical Package for the Social Sciences software, version 25 (IBM SPSS Statistics; SPSS Inc) and GraphPad Prism 8 (GraphPad Software, San Diego, CA, USA). Results were expressed as mean ± SEM or in fold change between conditions, calculated as mean fold change ratio (and deviation) in IBM patients versus CTL fibroblasts, normalized by cell number or by protein content. Obtained data were compared between independent experiments using the non‐parametric Mann–Whitney *U* test or Kruskal–Walli's test. Statistically significant results (*P*‐value < 0.05) were highlighted along the manuscript. Additionally, borderline significant results were included according to their biological relevance for better understanding of the biological context.

## Results

### Clinical and epidemiological data

In this study, IBM patients belonged to Caucasian population between 40 and 83 years old (mean age 66.9 ± 3.6) following a 1:1.7 male to female ratio (*n* = 14). Control subjects also belonged to the Caucasian population (*n* = 12), from 35 to 86 years old (mean age 57.4 ± 4.6), following a 1:2.5 male to female ratio. According to the IBM functional rating scale (IBMFRS; ranges from 0 (inability)‐40 (normal behaviour)^20^), the IBM cohort scored 25.1 ± 2.1, indicating moderate clinical severity. The average time from symptoms onset to diagnosis was 37 months; six patients showed stable IBM prognosis and eight of them had a progressive disease decline. This overview of clinical data is summarized in Table 1, together with patients' muscle biopsy features that aid in IBM diagnosis.

**Table 1:** Clinical data of the cohorts and features from IBM patients muscle's biopsies

Sample code (IBM)	Sex	Age	Time symptoms to diagnosis (months)	IBMFRS	Evolution	Rimmed vacuoles	Inflammation	MHC1	Auto Ab	CD57	Mitochondrial Changes	
IBM01	F	67	26	16	Stability	+	+	NA	All −	Normal	No	
IBM02	M	51	48	16	Progression	++	++	NA	All −	Increase	No	
IBM03	M	48	48	31	Stability	+	+	NA	All −	NA	Yes	
IBM04	F	81	26	17	Progression	+++	+	NA	All −	Normal	Yes+	
IBM05	M	63	36	27	Progression	+	+++	Universal	All −	NA	Yes +	
IBM06	M	77	48	28	Progression	+	+	Neg	All −	Increase	Yes	
IBM07	F	61	36	23	Progression	+++	+++	Universal	All −	NA	Yes	
IBM08	F	83	12	8	Progression	+	+++	NA	All −	T clonal	Yes +	
IBM09	M	62	84	30	Progression	+++	++	Universal	All −	Increase	Yes	
IBM10	M	78	12	28	Progression	+++	+++	+ Not universal	All −	NA	No	
IBM11	F	80	24	33	Stability	+++	+	Universal	Ro +	Normal	Yes +	
IBM12	M	71	36	30	Stability	+++	+++	Universal	Ro52+	Increase	Yes	
IBM13	M	75	72	32	Stability	+	++	Universal	All −	Reduced B normal CD57	Yes	
IBM14	M	40	3	33	Stability	+++	++	Universal	All −	NA	Yes+	
Sample code (CTL)	CTL01	CTL02	CTL03	CTL04	CTL05	CTL06	CTL07	CTL08	CTL09	CTL10	CTL11	CTL12
Sex	M	F	F	F	F	F	M	F	F	M	M	M
Age	69	66	61	41	48	47	35	45	82	86	57	52

Summary	Fibroblasts (*n*)		Male/female ratio			Mean age (years)		Time to diagnose (months)			IBMFRS	
IBM	14		0.6			66.9 ± 3.6		37			25.1 ± 2.1/40	
CTL	12		0.4			57.4 ± 4.6						

*Note*: Data presented as mean ± SEM.

Abbreviations: Sex: F, female; M, male; IBMFRS, Inclusion Body Myositis Functional Rating Scale (from 0 to 40 points).^20^ Rimmed vacuoles: from 0 to +++; Inflammation: from 0 to +++; MHC1: Major Histocompatibility Complex: Universal; NA: not available; + not universal: splattered pattern; Auto Ab: Autoantibodies: Myositis specific and associated auto antibodies include: Anti‐Jo‐1, anti‐SRP, anti‐Mi2, anti‐TIF1gamma, anti‐MDA5, anti‐SAE, anti‐NXP2 and anti‐HMGCR (specific) and anti‐PM/Scl, anti‐Ro, anti‐La, and anti‐U1RNP (associated); Mitochondrial changes: Yes or No. Yes: RRF and/or COX neg. Yes + (strong alterations). No: no changes; Age and gender‐paired distribution between cohorts (*P* = NS).

### Transcriptomic phenotyping: Altered gene expression in IBM fibroblasts

**Figure 1:**
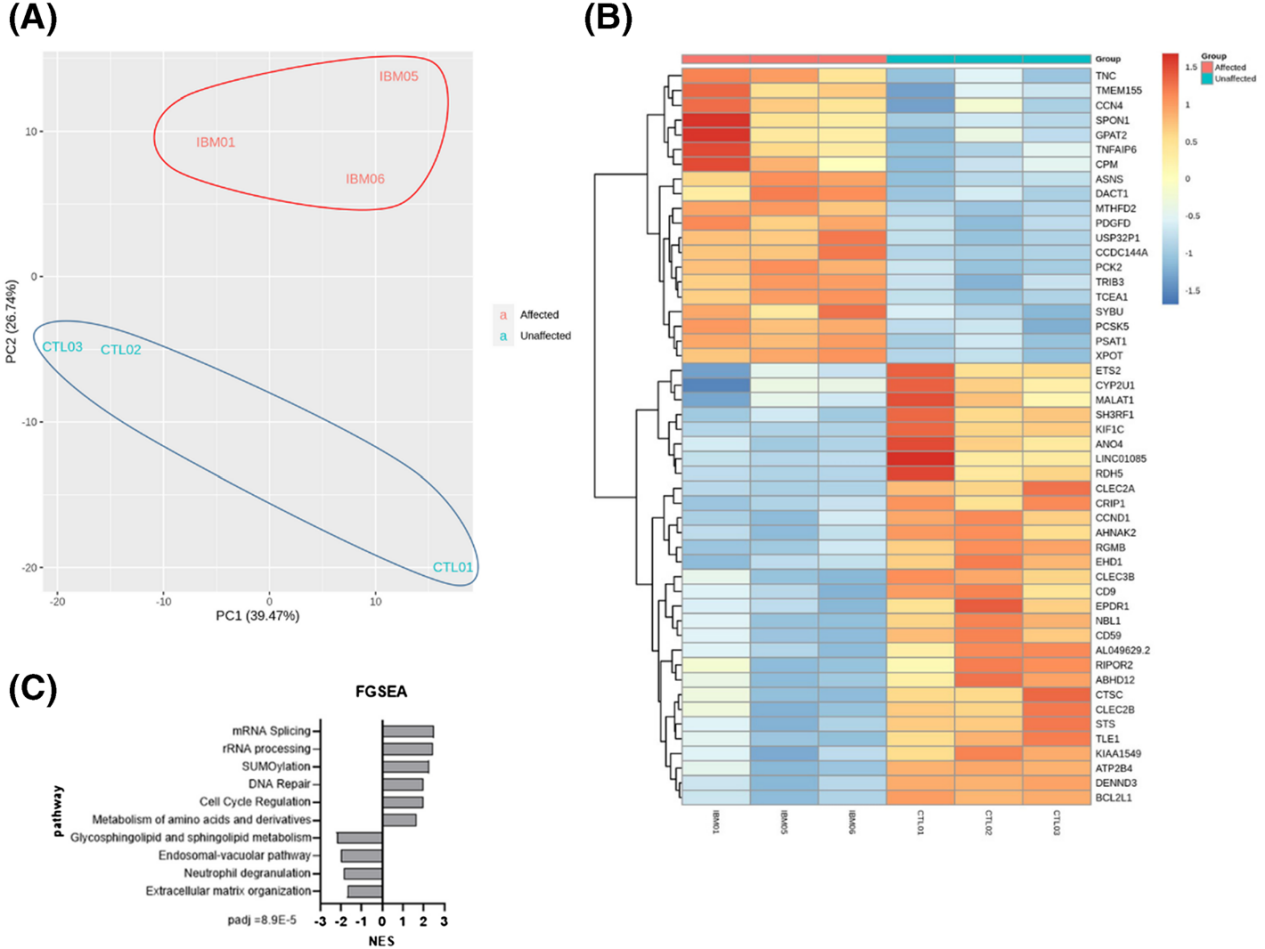
Transcriptome analysis of IBM and CTL fibroblasts (*n* = 3/group). (A) Principal component analysis (PCA) of affected (IBM) versus unaffected (CTL) subjects. (B) Heatmap of the top 50 differentially expressed genes (DEGs) obtained in a differential expression analysis between both cohorts. All DEGs are represented in *Table* S1. (C) Top pathways (with lowest *P*‐value adj) after a functional gene set enrichment analysis (GSEA) and considering their normalized enrichment score (NES). PCA, heatmap and pathway analysis revealed a differential gene expression pattern in IBM patients, with DEGs and pathways related to inflammation, mitochondria and other processes like cell cycle regulation and metabolism of amino acids and derivatives.

RNA sequencing was performed in fibroblasts from IBM patients (*n* = 3) and genetically unrelated healthy subjects (*n* = 3). A principal component analysis (PCA) revealed IBM and healthy subjects clustered separately (*Figure* 1A), suggesting a differential gene expression pattern of disease, comprising 778 DEGs with *P*‐value adj (FDR) < 0.05; of them, 457 were downregulated and 321 upregulated (*Table* S1). The top 50 DEGs are represented in the heatmap (*Figure* 1B). Among these DEGs, some are related to inflammation (MHC components like *CD59*; T‐cell expansion genes like *CLEC2A*, *CLEC2B*, and *CLEC3B*, and others like *CSCS* and *TNFAIP6*); mitochondria (*PCK2*, *MTHFD2*, *GPAT2*, *ATP2B4*, and *CYP2U1*); and other processes like cell cycle regulation, metabolism of amino acids and derivatives, and so on. Gene names are found in *Table* S1.

These DEGs were clustered with a Gene Ontology enrichment analysis, where the most deregulated clusters were related to *cell communication*, *cell signalling* and *systems development*. Gene set enrichment analysis (GSEA) revealed 358 significantly altered pathways (*P*‐value < 0.05), the top ones found in *Figure* 1C.

In addition to GSEA, DEGs were analysed using Ingenuity Pathway Analysis (IPA) software. IPA showed *tRNA charging* as the top canonical pathway, top networks related to *cell cycle*, *DNA replication*, *cell‐to‐cell* signalling, and *interaction*, similar to GSEA results, confirming the consistency of reported findings. Overall, these findings point out a clear deregulated transcriptomic profile in IBM fibroblasts and highlight significant pathways and molecular targets in IBM.

### Functional phenotyping

#### Increased inflammatory profile in IBM fibroblasts

Inflammation is one of the hallmarks of IBM in muscle. To explore inflammation in IBM versus CTL fibroblasts, we performed an mRNA‐seq and measured secreted cytokines in cell supernatants.

**Figure 2:**
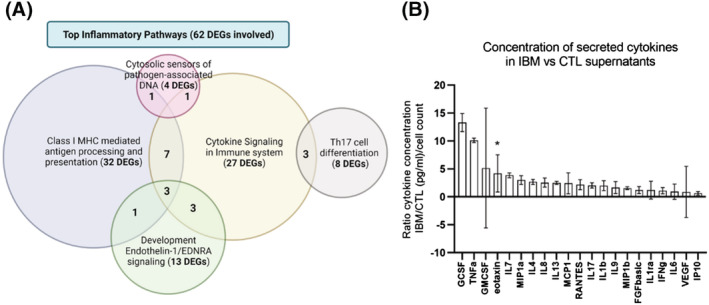
The inflammatory pattern of IBM and CTL fibroblasts. (A) Venn's diagram of the most affected inflammatory clusters and the number of differentially expressed genes (DEGs) involved. Some DEGs are involved in more than one cluster. The list of inflammatory DEGs is represented in *Table* S2. (B) Mean fold change ratio (and deviation) of secreted cytokines in IBM patients versus CTL fibroblasts, normalized by cell number (*n* = 13 IBM vs. 12 CTL; *P‐value < 0.05, Mann–Whitney *U* test) (ratio > 1: Higher concentration in IBM). The concentration of each cytokine and their full names are represented in *Table* S3. Briefly, an increased inflammatory profile is observed in IBM versus CTL fibroblasts, with differentially expressed genes (DEGs) related to MHC1, cytokines, interleukins, and so on and higher cytokine secretion in fibroblast supernatants.

In the transcriptome, we found a differential inflammatory profile in IBM versus CTL fibroblasts. Briefly, we found 62 inflammatory‐related genes (*Table* S2) related to MHC1, cytokines and IL receptors, NF‐kB pathway, IFN genes and T‐cell expansion genes, which were grouped in gene clusters (*Figure* 2A), all involved in IBM pathogenesis.

Considering cytokine secretion in fibroblast supernatants, we quantified 21 pro‐inflammatory cytokines, grouped in chemokines (eotaxin, IP10, MCP1, MIP1a, MIP1b, RANTES, and IL8) and proinflammatory cytokines (GCSF, TNFa, GMCSF, IL7, IL4, IL13, IL17, IL1b, IL9, FGFb, IL1ra, IFNg, IL6, and VEGF). Cytokines drifted towards an upregulated three‐fold expression in IBM patients (*Figure* 2B and *Table* S3). In particular, eotaxin (*CCL11*), involved in a chemotactic activity for eosinophils,^21^ showed a statistically significant increase of 4.2 times in IBM versus CTL fibroblasts (*P*‐value < 0.05, *n* = 13 IBM vs. 12 CTL), whereas GCSF (that regulate the production, differentiation, and function of granulocytes) showed a high 13.3‐fold change ratio between groups (*Figure* 2B). These findings revealed a trend towards an increased inflammation in IBM fibroblasts at a transcriptomic and functional level, identifying relevant pathways and targets in this pathology.

#### Degenerative features and impaired autophagy in IBM fibroblasts

Rimmed vacuoles are another hallmark in the muscle of IBM patients, partly due to misfolded protein depots in the cytoplasm of muscle cells, caused by impaired or insufficient autophagy. Here, we examined autophagy in IBM versus CTL fibroblasts by mRNA‐seq data and analysing target proteins at basal and time‐course manners.

**Figure 3:**
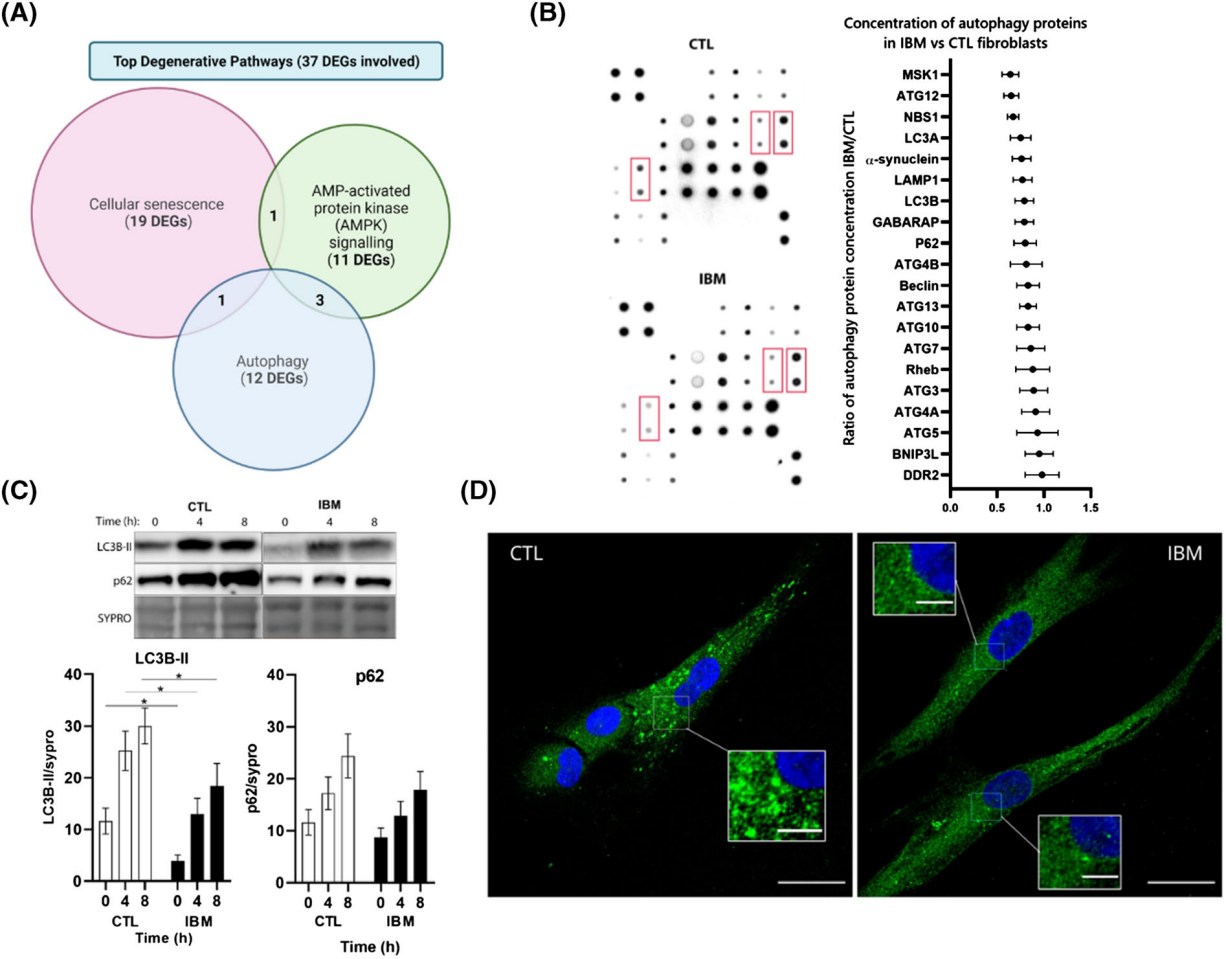
Autophagy in IBM versus CTL fibroblasts. (A) Venn's diagram of the most affected degenerative clusters and the number of differentially expressed genes (DEGs) involved. Some DEGs are involved in more than one cluster. The list of degenerative DEGs is represented in *Table* S2. (B) Representative images of two autophagy protein arrays of one inclusion body myositis (IBM) and one control (CTL) fibroblast and quantification of *n* = 8/group autophagy protein arrays. In red, differences in protein concentration (according to spot intensity) among IBM and CTL fibroblasts. In the graph, mean fold change ratio (and deviation) of autophagy proteins in IBM patients versus CTL fibroblasts, normalized by protein content (ratio < 1: Lower concentration in IBM). Each protein concentration is depicted in *Table* S4. (C) Representative images and graphs of an autophagy time‐course (at basal, 4 h and 8 h under bafilomycin A1 treatment) measured by western blot between IBM (*n* = 13) and CTL (*n* = 12) fibroblasts. Results: Mean ± SEM, *n* = 13 IBM versus 11 CTL; Kruskal–Wallis test, *P‐value < 0.05. SQSTM1/p62: Autophagy substrate, LC3BII: Autophagy receptor, lipidated form, sypro: Total protein content. (D) Representative images of autophagosome accumulation in IBM versus CTL fibroblasts treated with bafilomycin A1 (100 nM) for 6 h obtained by confocal microscope (LC3B in green; nuclei in blue with DAPI, scale 25 μM (inserts 5 μM)). Briefly, autophagy is impaired in terms of differentially expressed genes (DEGs) and decreased expression of basal protein mediators, time‐course autophagosome formation, and microscopic evaluation.

In the gene expression analysis, we found 37 DEGs related to autophagy (*Table* S2) affecting many steps of the signalling cascade (heat‐shock proteins, stress‐related factors, proteasome genes and regulators of the master *AMPK* gene), which were grouped in degenerative gene clusters (*Figure* 3A).

In the functional autophagy analysis, we assessed expression of 20 proteins at steady‐state levels in IBM and CTL fibroblasts. The expression of autophagic proteins was 18.4% reduced in IBM (*Table* S4 and *Figure* 3B), leading towards a downregulation of the whole autophagic process. In particular, the following pathways were more affected^22^: *Receptor‐mediated mitophagy* (ATG5, ATG12, LC3A, and LC3B), *PINK1‐PRKN Mediated Mitophagy* (ATG12, P62, LC3A, LC3B, and ATG5) and the super pathway *Macroautophagy* (ATG12, ATG7, Rheb, ATG4A, ATG13, Beclin, P62, GABARAP, LC3B, ATG5, ATG10, LC3A, ATG3, and ATG4B) that displayed a trend of 22.0%, 21.6%, and 17.5% reduction in IBM fibroblasts.

To evaluate if a decreased autophagic activity was due to the inactivation of the process or, to higher consumption of mediators caused by a higher autophagic rate, we performed a time‐course experiment that blocked the process. As observed in *Figure* 3C, flux rate of both LC3BII and p62 effectors at 4 h and 8 h after bafilomycin A1 treatment was non‐significantly reduced between cohorts when normalized by basal levels. Interestingly, raw data non‐normalized by basal expression showed significantly reduced levels of LC3B‐II marker at 4 h and 8 h after treatment in IBM fibroblasts (44.4% and 33.6% reduced in IBM, *P*‐value < 0.05), suggesting a lower autophagic performance in the IBM cohort. Overall, a decreased autophagic activity could lead to insufficient cell recycling and accumulation of damaged components in the cytoplasm, increasing the endoplasmic reticulum stress and compromising the homeostatic balance within cells. In the case of p62 expression, we also found a slight reduction in IBM fibroblasts (24.9%, 19.0%, and 20.6% reduced).

To visualize the proportion of autophagic vesicles between cohorts, we performed immunostaining of LC3B after 6 h under bafilomycin A1 treatment (*Figure* 3D). The results revealed a lower accumulation of autophagosomes in IBM fibroblasts. These findings manifested a deregulated autophagic and degenerative profile in IBM fibroblasts, prompting relevant pathways and targets in this disease.

#### Mitochondrial abnormalities increased oxidative status in IBM fibroblasts

The third hallmark of IBM in muscle are mitochondrial alterations. In muscle biopsies, there is an increased number of mitochondria across myofibres (creating RRF) and increased complex II expression (SDH+ fibres), probably as an attempt to compensate dysfunctional mitochondria, evidenced as decreased complex IV expression (COX‐fibres).^6^, ^11^, ^23^, ^24^ These are features of primary mitochondrial diseases. We aimed to examine the mitochondrial state of IBM versus CTL fibroblasts by analysing mitochondrial deregulated genes, and by functional assessment of bioenergetic and metabolic performance.

**Figure 4:**
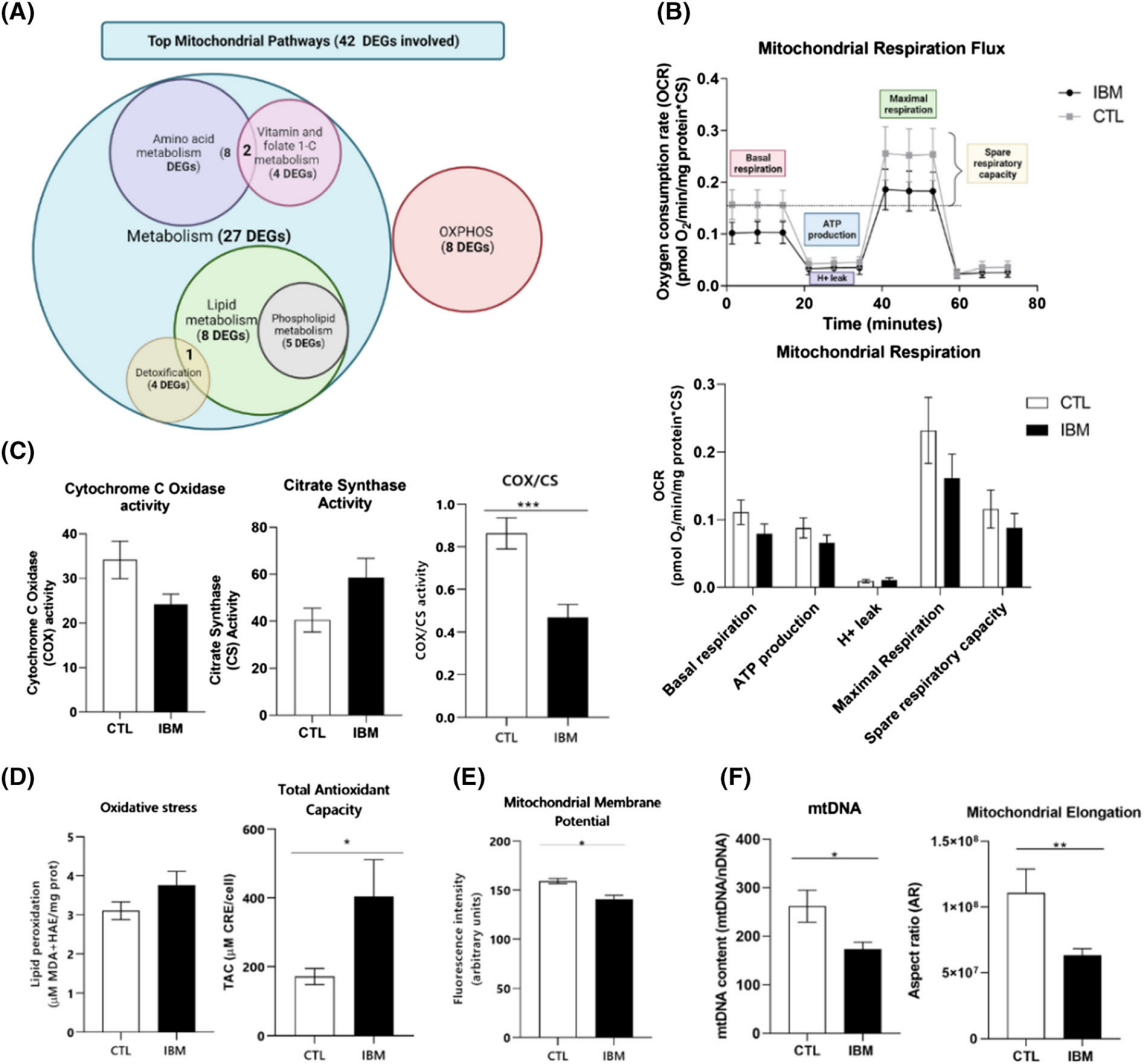
Mitochondrial profile in IBM versus CTL fibroblasts. (A) Venn's diagram of the most affected mitochondrial clusters and the number of differentially expressed genes (DEGs) involved. Many DEGs are involved in more than one cluster. The list of mitochondrial DEGs is represented in *Table* S2. (B) Mitochondrial respiratory flux and rates of IBM versus CTL fibroblasts. Each sample was seeded in quadruplicate per condition (*n* = 10/group, Mann–Whitney *U* test). OCR = oxygen consumption rate. Respiratory control ratios were normalized by total protein content and by citrate synthase (CS) activity as a marker of mitochondrial content. (C) Cytochrome C oxidase (COX), citrate synthase (CS) and COX per CS activity (COX/CS ratio, as a marker of complex IV from the mitochondrial respiratory chain (MRC) per mitochondrial mass (*n* = 10/group, *P*‐value < 0.001, Mann–Whitney *U* test), normalized by protein content. (D) Oxidative stress measured through lipid peroxidation in IBM versus CTL fibroblasts normalized by total protein content (*n* = 9 IBM vs. 8 CTL, Mann–Whitney *U* test) (MDA: Malondialdehyde; HAE: Hydroxialkenal); coupled to Total antioxidant capacity (TAC) expressed as μM CRE (copper reducing equivalents) per total cell number (*n* = 11 IBM vs. 4 CTL, *P*‐value < 0.05, Mann–Whitney *U* test). (E) Mitochondrial membrane potential (MMP) measured with TMRM + Arimoclomol in IBM versus CTL fibroblasts (*n* = 4/group, *P*‐value < 0.05, Mann–Whitney *U* test). The results were expressed as mean ± SEM (*P < 0.05). (F) mtDNA genome content in IBM versus CTL fibroblasts expressed as the ratio of mtDNA 12S rRNA:nDNA RNase‐P (mitochondrial vs. nuclear encoded gene) (*n* = 9 IBM vs. 8 CTL, *P*‐value < 0.05, Mann–Whitney *U* test) and mitochondrial elongation (represented by the aspect ratio) in IBM versus CTL fibroblasts (*n* = 3/group, *P*‐value < 0.05, Mann–Whitney *U* test). Briefly, mitochondrial performance is disturbed in IBM versus CTL fibroblasts in terms of genetic content, differentially expressed genes (DEGs) and functionality (reduced respiratory profile, marked COX/CS decline, higher oxidative stress and antioxidant defence activation, reduced MMP and mitochondrial elongation).

Regarding mitochondrial gene expression, we found 42 mitochondrial DEGs compiled in the Mitocarta (version 3.0),^25^ in *Table* S2. Among them, some DEGs were encoded in the mitochondrial genome (NADH:CoQ oxidoreductase subunits, COX and ATP synthase), others related to the MRC function but encoded in the nuclear genome; and tricarboxylic acid cycle (TCA) enzymes. Also, some DEGs were stress‐related or peroxisomal genes. These genes were grouped in gene clusters (*Figure* 4A).

At functional level, we measured the respiratory profile of IBM and CTL fibroblasts to quantify the oxygen consumption rate (*Figure* 4B). IBM fibroblasts leaned towards a decreased respiratory pattern (*Figure* 4B), especially at basal and maximal respiration levels (28.6% and 31.8% reduced in IBM), suggesting a trend towards less active MRC activity.

These results were supported at the enzymatic level by a 45.6% COX significant decrease, as seen in muscle, normalized by CS activity (*P*‐value < 0.001) (*Figure* 4C). Moreover, CS activity shifted towards a 44.6% increase in IBM fibroblasts, leading to an increased mitochondrial mass to compensate for COX reduction, as seen in RRF from IBM muscles.

To assess if MRC downward is correlated to oxidative stress and antioxidant levels, lipid peroxidation markers like MDA and HAE were measured in cell lysates (*Figure* 4D). Lipid peroxidation tended to increase a 14.3% in IBM versus CTL fibroblasts, suggesting a trend towards higher oxidative stress in IBM cells; coupled with a 135.2% significant increased level of antioxidant defences (TAC) in IBM (*P*‐value < 0.05) (*Figure* 4D). Moreover, in the mRNA‐seq we found aldehyde dehydrogenases (*ALDH1L2*, *ALDH1B1*, *ALDH4A1*, and *ALDH18A1*), enzymes involved in lipid peroxidation, and some antioxidant enzymes (*CBR3*, *CYB5R3*, *ETHE1*, *PRXL2A*, and *SQOR*), among the DEGs between IBM and CTL fibroblasts.

Mitochondrial dysfunction is usually linked to the depolarization of MMP, that was significantly decreased by 11.6% in IBM fibroblasts (*P*‐value < 0.05) (*Figure* 4E). MMP depolarization may result in impaired proton pumping, leading to the opening of the mitochondrial membrane pore, and potential activation of apoptosis.

**Figure 5:**
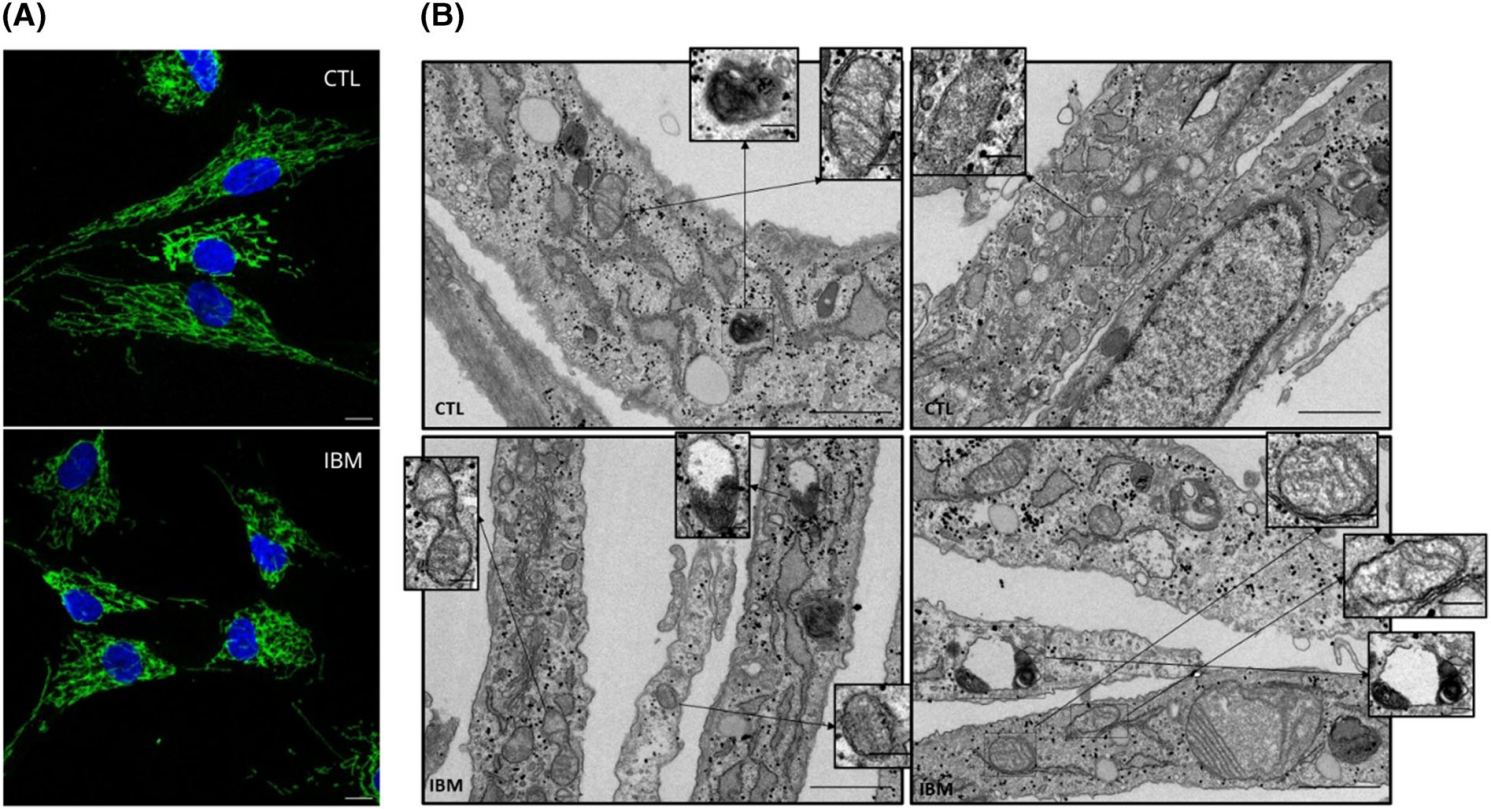
Mitochondrial morphology in IBM versus CTL fibroblasts. (A) Mitochondrial network in a representative image of IBM versus CTL fibroblasts obtained by confocal microscope (TOMM20 in green; nuclei in blue with DAPI, scale 10 μM) displaying reduced mitochondrial elongation in IBM (associated to poor mitochondrial health). (B) Transmission electron microscopy (TEM) images of IBM versus CTL fibroblasts. In IBM fibroblasts, some mitochondria lacked cristae (see inserts) presenting a discontinuous cristae distribution. Also, increased mitochondrial fission (see insert) could explain the smaller and more circular shape of these organelles associated with unhealthier mitochondria. Additionally, the presence of impaired autophagosome‐lysosome structures in IBM patients (see inserts) might be a result of incomplete autophagy. Briefly, these findings highlight a mitochondrial impairment in IBM fibroblasts. Magnification: 30 000×, scale 1 μm for each picture and 0.2 μm for inserts.

mtDNA genome content was significantly decreased by 33.9% in IBM fibroblasts (*Figure* 4F; *P*‐value < 0.05), as previously observed in IBM muscle^11^ and in accordance to abnormal mitochondrial morphology, either externally (showing a significant 42.8%‐reduced mitochondrial elongation, *Figure* 4F, *Figure* 5A), and internally (at subcellular level) (*Figure* 5B). Briefly, using TEM ultrastructural visualization, 76.2% of IBM fibroblasts revealed abnormal mitochondria (61.9% of them with altered cristae and 42.2% with a round shape, associated with unhealthier mitochondria), compared with 25.1% of CTL fibroblasts with abnormal mitochondria (of which 7.9% had altered cristae and 17.2% a round shape). At mRNA level, we found three DEGs related to mitochondrial dynamics (BCL2L1, FKBP8, and MGARP), all downregulated.

Overall, these findings highlight a mitochondrial impairment in IBM fibroblasts and identify contributing pathways and key players in disease.

#### Targeted metabolites characterization

To examine if mitochondrial dysfunction was linked to metabolic misbalances beyond the MRC, we measured DEGs and metabolite levels related to organic acids and amino acids in IBM and CTL fibroblasts.

At gene expression level, we observed 10 DEGs involved in organic acids and amino acids metabolism (in *Table* S5). Briefly, increased lactic acid and alanine are related to *MT‐ND5*, *MT‐CO2*, and *MT‐ATP6*, components of the MRC and related to the TCA cycle. *ETHE1* is also linked to increased alanine, ethylmalonic and methyl succinic, and related to the *SQOR* gene. *PSAT1* and *GPX3*, albeit not being mitochondrial genes, are directly related to mitochondrial metabolism, involved in serine and glycine biosynthesis and pyroglutamic acid metabolism, respectively.

**Figure 6:**
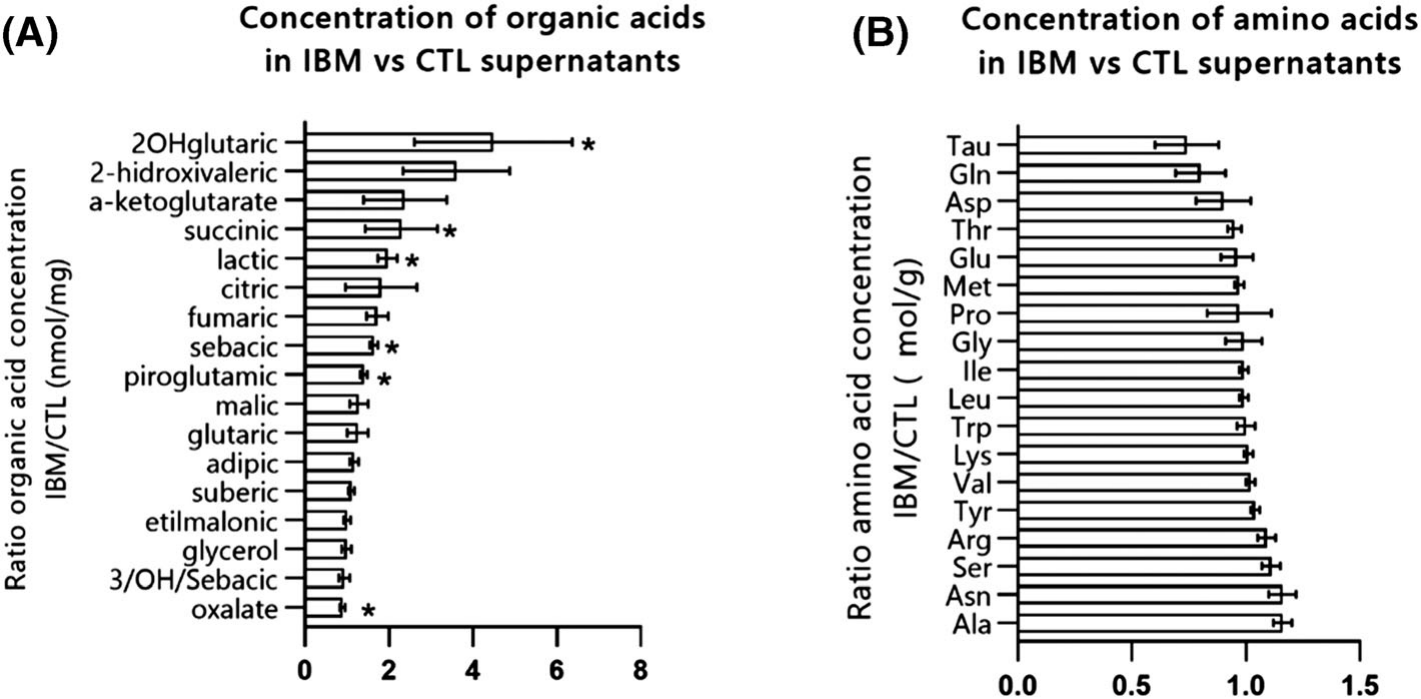
Metabolites concentration in IBM versus CTL fibroblasts. (A) Mean fold change ratio (and deviation) of organic acids in IBM patients versus CTL fibroblasts, normalized by protein content (*n* = 11 IBM vs. 10 CTL, *P‐value < 0.05, Mann–Whitney *U* test) (ratio > 1: Higher concentration in IBM). The raw data of each organic acid concentration is represented in *Table* S6. (B) Mean fold change ratio (and deviation) of amino acids IBM patients versus CTL fibroblasts, normalized by protein content (*n* = 11 IBM vs. 10 CTL, Mann–Whitney *U* test) (ratio > 1: Higher concentration in IBM; ratio < 1: Lower concentration in IBM). The raw data of each amino acid concentration is represented in *Table* S7. Briefly, organic acid levels showed a general increase in IBM, suggesting deregulation of intermediary metabolism related to mitochondrial dysfunction, whereas no relevant differences in amino acid levels were observed between IBM and CTL fibroblasts.

At functional level, organic acids showed a 1.8‐fold change increase in IBM, suggesting deregulation of intermediary metabolism. The most relevant organic acids were: lactic acid (biomarker of mitochondrial diseases); succinic acid (part of MRC complex II, increased in SDH+ fibres in muscle); pyroglutamic acid (involved in glutathione synthesis); sebacic acid (involved in the electron transfer from the β‐oxidation pathway to MRC complex II) and above all, 2OH‐glutaric acid (TCA cycle component which accumulates in case of mitochondrial defect), that were significantly deregulated (*Figure* 6A and *Table* S6). These components or derivatives of the TCA cycle are related to amino acids or fatty acid metabolism and their increase is frequently linked to MRC dysfunction. Regarding amino acids levels, no relevant differences were observed between IBM and CTL fibroblasts (*Figure* 6B and *Table* S7).

Metabolic reprogramming in IBM fibroblasts supports mitochondrial dysfunction beyond the MRC, and unravels the main pathways and targets involved in IBM.

#### Association of molecular phenotype and disease progression

Phenotyping IBM vs CTL fibroblasts has revealed an overall differential gene expression and functional profile, especially considering inflammation, degeneration and mitochondrial function. Interestingly, this pattern seems to differ slightly depending on disease evolution.

**Figure 7:**
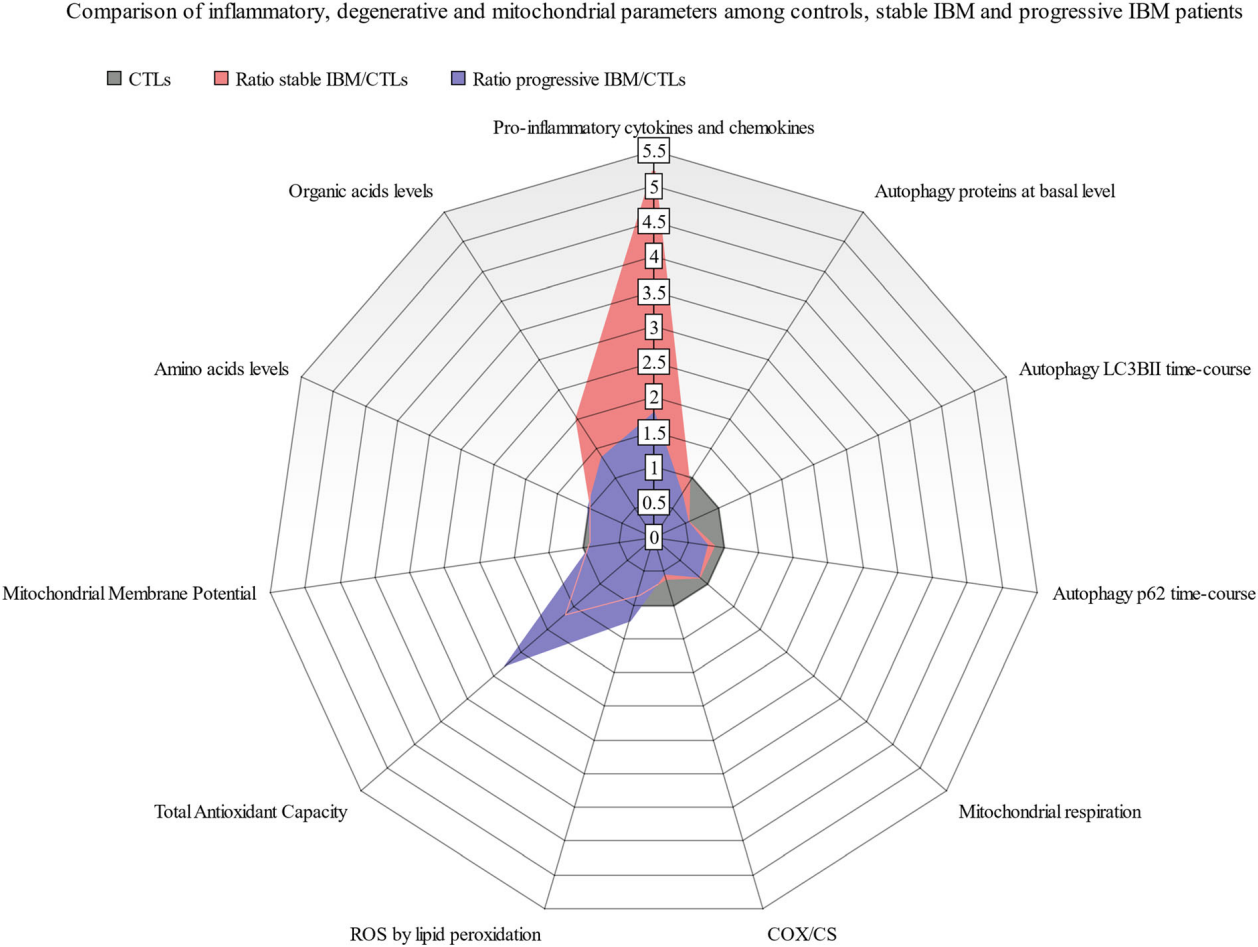
Comparison of inflammatory, degenerative and mitochondrial parameters among controls (CTLs), IBM patients with stable evolution (stable IBM) and IBM patients with disease progression (progressive IBM). Ratios between IBM (stable or progressive) and control fibroblasts were measured to compare inflammatory, degenerative and mitochondrial parameters among the three groups (*Table* S8). Briefly, stable IBM showed an increased pro‐inflammatory and organic acids signature compared with progressive IBM and controls. Progressive IBM showed increased oxidative stress (ROS) and antioxidant capacity. Together, IBM stable and progressive present an evident decrease in LC3BII time‐course (autophagy marker) and COX/CS (cytochrome c oxidase related to citrate synthase, a marker of mitochondrial mass) with respect to CTLs (ratio > 1: Higher concentration in IBM; ratio < 1: Lower concentration in IBM).

As observed in Table 1, six patients showed stable IBM prognosis whereas eight followed a progressive disease decline. The correlation of molecular and clinical data yielded to the association of increased pro‐inflammatory and organic acids signature in stable IBM (*Table* S8 and *Figure* 7). Contrarily, progressive IBM showed increased oxidative stress and antioxidant capacity. Together, stable and progressive IBM present an evident decrease in LC3BII time‐course and COX/CS with respect to CTLs.^26^

The presence of common and differential molecular profile between stable and progressive IBM patients could help in understanding disease pathogenesis and become potential markers of prognosis.

## Discussion

Here we reported the validation of fibroblasts as a disease model for untangling IBM and identifying novel pathogenic molecular players. Briefly, we observed transcriptomic and functional inflammatory, degenerative and mitochondrial changes in IBM fibroblasts, which resemble the alterations found in the target tissue, in muscle.

Previous studies analysed IBM transcriptome^27^, ^28^ at muscular and blood level, but this is the first IBM RNA‐seq in fibroblasts. Here we found 778 DEGs, supporting a clear pathological pattern of gene expression in IBM.

At the inflammatory level, IBM fibroblast transcriptome revealed altered inflammatory genes resembling muscle features, including MHC1 (*HLA* genes) and *IFN* genes. These alterations were found in an IBM deep neural network model.^29^, ^30^ At the functional scale, cytokines secretion in IBM fibroblasts was increased. Previous studies in IBM muscle detected CD8+ T cell‐secreted cytokines and chemokines related to IFN‐ɣ genes (*CXCL9*, *CXCL10*, *CCL5*, and *CCL18*) and others, including *TNF*, *IL7*, *IL15*, *IL16*, and *IL32*,^1^, ^26^ some of which were also detected in the sera of IBM patients.^26^ Although fibroblasts are not part of the immune system, these results partially mimic the inflammation of IBM muscles.

Regarding degeneration in IBM, impaired autophagy and disturbed proteostasis have been described in muscle.^24^ Some degenerative markers like TDP43, p62, and LC3 show an aberrant expression in IBM muscle.^5^, ^31^ Herein, we found impaired autophagy at gene and functional level, with lower production of autophagy proteins and deficient formation of autophagosomes in IBM fibroblasts, leading to insufficient recycling needs and deposition of cargos within the cell. This phenotype is remarkable in fibroblasts, as their high turnover avoids debris accumulation. In contrast, muscle is a post‐mitotic tissue that accumulates cell debris, forming rimmed vacuoles in IBM, where autophagic deficiencies are more evident.

Related to deficient macroautophagy and mitophagy in IBM, there is a lower turnover of mitochondria, leading to bioenergetic deficits and cell damage due to oxidative stress generation.^24^, ^32^ The deficient mitochondrial profile observed in IBM fibroblasts revealed altered gene expression, impaired respiratory capacity, increased oxidative stress and mitochondrial mass (CS), reduced MMP, COX activity, mtDNA content and mitochondrial elongation, all previously seen in muscle tissue^11^, ^13^, ^24^ and in mitochondrial myopathies,^13^, ^33^ suggesting that IBM slightly behaves as a mitochondrial disease.

Metabolic reprogramming could be linked to mitochondrial dysfunction, as proposed in IBM blood metabolomes^33^ and primary mitochondrial diseases. Many pathways and genes related to the IBM blood metabolome are altered in IBM fibroblasts. Buzkova *et al*. demonstrated that IBM blood metabolome clustered more accurately with mitochondrial myopathies than neuromuscular diseases,^33^ prompting a higher mitochondrial implication in IBM than previously thought. The current results in fibroblasts support previous findings.

Interestingly, these pathological features of IBM fibroblasts and muscle (inflammation, degeneration and mitochondrial dysfunction), resemble standard physiologic aging, including decreased muscle strength,^23^ supporting that IBM involves an accelerated muscle aging.

Considering IBM evolution, it revealed that progressive IBM (22.1 ± 2.7 IBMFRS) is associated to higher imbalances in oxidant and antioxidant capacities whereas stable IBM (29.2 ± 2.7 IBMFRS) is associated to higher inflammatory profile. This increased inflammation could be a protective mechanism in the stable IBM, as these patients perform better at a clinical scale (higher IBMFRS). Considering these findings, oxidative stress and inflammation emerge as potential markers of prognosis.

Overall, this is the first attempt to suggest fibroblasts as a model of IBM pathogenesis, scarcely tested as a model in neuromuscular diseases.^12^, ^34^ So far, most of the pathological mechanisms in IBM have been discovered in cells and tissue samples from patients. However, although preclinical and omic studies help in understanding disease pathogenesis, the gap in translation to clinical research remains elusive.^35^ Currently in IBM, 16 clinical trials are active,^36^ in which most treatments were discovered or tested in cell models (e.g. phenylbutyrate in *in vitro* muscle fibres^37^), suggesting the relevance of disease models in regulatory settings. In this sense, fibroblasts emerge as a promising model of IBM to discover new targets and perform screening of biomarkers and potential therapies.

Nevertheless, there are some limitations in the present study. First, our limited sample size, mainly due to IBM being a rare disease, even though for a reference unit like ours. In addition, even fibroblasts are associated to cost‐effective maintenance conditions, other cell models, closer to the target tissue, might better reflect IBM pathogenesis. This may be the case of myotubes differentiated from induced pluripotent stem cells, which avoid the invasiveness of a muscle biopsy, but may offer a closer approach to muscle behaviour. However, they require complex protocols, rending fibroblasts to a cost‐effective cell model.

Despite these contingencies, in the present study we validated fibroblasts as a disease model for IBM, showing IBM alterations are present beyond the target tissue of the disease. This model could be exported to other myositis or neuromuscular diseases. Additionally, we unveil numerous pathways and specific molecules that emerge as relevant key players in disease aetiology or evolution, for further evaluation as candidate biomarkers or therapeutic targets.

## Funding

This study was supported by Instituto de Salud Carlos III (ISCIII‐FEDER, grants PI18/00498, PI18/00451 and PI21/00935) and co‐funded by the European Union. Additionally, JC‐S is supported by the APIF Programme (University of Barcelona), FJG‐G by the CIBERER (ISCIII‐FEDER), MG‐M by CD21/00019 (ISCIII‐ FSE+), CR by the Serra Húnter Programme (Generalitat de Catalunya), RA by Torrons Vicens, JCF‐C by PID2019‐111669RB‐I00, CG‐R by PID2020‐115055RB‐I00 and AE‐C by PT17/0009/0019 (ISCIII‐MINECO‐FEDER).

## Conflict of interest

The authors declare that they have no conflict of interest.

## Supporting information


**Table S1.** Differentially expressed genes between IBM and CTL (778 genes with p‐value adj (FDR) < 0.05, following alphabetical order)
Table S2. Lists of differentially expressed genes (DEGs) related to inflammation (62 DEGs), autophagy (37 DEGs) and mitochondria (42 DEGs)
Table S3. Concentration of secreted inflammatory cytokines in IBM vs CTL supernatants. IBM fibroblasts revealed an increased expression of most of these cytokines.
Table S4. Expression of 20 autophagy proteins in IBM vs. CTL fibroblasts. Most of these proteins displayed a decreased expression in IBM, suggesting a reduced activity of the autophagy process.
Table S5. Differentially expressed genes (DEGs) related to metabolite concentration in fibroblasts. The table depicts the relationship of 10 DEGs involved in metabolite metabolism with their respective alteration at amino acids and organic acids level, to relate expression vs. metabolism patterns in IBM vs. CTL fibroblasts.
Table S6. Organic acids in IBM vs. CTL fibroblasts. Organic acids showed a general increase in IBM, suggesting a deregulation of intermediary metabolism related to mitochondrial function, as many organic acids are involved in tricarboxylic acid cycle (TCA) that further feeds the mitochondrial respiratory chain.
Table S7. Amino acids levels in fibroblasts of IBM patients vs. CTL fibroblasts.
Table S8. Comparison of inflammatory, degenerative, and mitochondrial IBM hallmarks considering the evolution of IBM patients: stable vs progressive prognosis, compared to CTL fibroblasts.
