# Supplementary material for: Unravelling inclusion body myositis using a patient‐derived fibroblast model
Source: J Cachexia Sarcopenia Muscle. 2023 Mar 1;14(2):964–77. doi: 10.1002/jcsm.13178 (PMC10067507; doi:10.1002/jcsm.13178)
Supplement: Supplementary file 1 — Table S1. Differentially expressed genes between IBM and CTL (778 genes with p‐value adj (FDR) < 0.05, following alphabetical order) Table S2. Lists of differentially expressed genes (DEGs) related to inflammation (62 DEGs), autophagy (37 DEGs) and mitochondria (42 DEGs) Table S3. Concentration of secreted inflammatory cytokines in IBM vs CTL supernatants. IBM fibroblasts revealed an increased expression of most of these cytokines. Table S4. Expression of 20 autophagy proteins in IBM vs. CTL fibroblasts. Most of these proteins displayed a decreased expression in IBM, suggesting a reduced activity of the autophagy process. Table S5. Differentially expressed genes (DEGs) related to metabolite concentration in fibroblasts. The table depicts the relationship of 10 DEGs involved in metabolite metabolism with their respective alteration at amino acids and organic acids level, to relate expression vs. metabolism patterns in IBM vs. CTL fibroblasts. Table S6. Organic acids in IBM vs. CTL fibroblasts. Organic acids showed a general increase in IBM, suggesting a deregulation of intermediary metabolism related to mitochondrial function, as many organic acids are involved in tricarboxylic acid cycle (TCA) that further feeds the mitochondrial respiratory chain. Table S7. Amino acids levels in fibroblasts of IBM patients vs. CTL fibroblasts. Table S8. Comparison of inflammatory, degenerative, and mitochondrial IBM hallmarks considering the evolution of IBM patients: stable vs progressive prognosis, compared to CTL fibroblasts. [file JCSM-14-964-s001.docx]

**SUPPLEMENTARY MATERIALS**

***SUPPLEMENTARY METHODS***

**Study design and population**

A case-control study was conducted from 2018 to 2022, in the Department of Internal Medicine from Hospital Clínic of Barcelona (Barcelona, Spain), including 14 patients and 12 healthy volunteers. IBM patients were diagnosed according to clinical and pathological tests performed in our hospital, after fulfilling the criteria proposed by the European Neuromuscular Centre for IBM diagnosis[1]. Exclusion criteria were: age < 40 years, family history of hereditary mitochondrial disease, comorbidities and concomitant infections or drug abuse. Fourteen IBM patients decided to participate in the present study and signed the informed consent previously approved by the Ethical Committee of our hospital (code HCB/2015/0562). At the time of inclusion, the severity of disease was evaluated in IBM patients according to the inclusion body myositis functional rating scale (IBMFRS), internationally validated to functionally evaluate disease-specific disability. Epidemiological data (age, gender, and ethnicity) was also compiled. Controls were collected from healthy volunteers, age and gender-paired to IBM patients, after excluding any comorbidity or pathological process, and signed the corresponding informed consent.

**Fibroblast culture**

Fibroblasts were obtained from a skin punch biopsy from cases and controls. Cells were grown in 25 mM glucose DMEM supplemented with 10% FBS and 1% penicillin-streptomycin, at 37 ◦C, in a humidified 5% CO_2_ air incubator (all from Gibco, Waltham, MA, USA). Cells were harvested and collected by trypsin (Gibco, Waltham, MA, USA), at 80% of confluence, and phenotyped at passages 3 to 10. Transcriptomic and functional analyses were performed from these fibroblasts.

**RNA extraction and mRNA library preparation and sequencing**

Total RNA was isolated from cell lysates (2 million of cells) of 3 IBM and 3 CTL fibroblasts using RNeasy Mini Kit (Qiagen, Hilden, Germany), according to the manufacturer’s protocol. Total RNA content was assessed through Quawell UV-Vis Spectrophotometer Q5000. Concentrations were adjusted to 100 ng/uL in RNase-free water. The quality control of the total RNA was done using the Qubit® RNA HS Assay (Life Technologies) and RNA 6000 Nano Assay on a Bioanalyzer 2100 (Agilent, Santa Clara, CA, USA). The RNASeq libraries were prepared using the TruSeq®Stranded mRNA LT Sample Prep Kit. Briefly, total RNA (500ng) was enriched for the mRNA fraction and fragmented. Strand-specificity was achieved by the second-strand cDNA incorporating dUTPs instead of dTTPs. The blunt-ended double stranded cDNA was 3´adenylated and Illumina platform compatible adaptors with unique dual indexes and unique molecular identifiers (Integrated DNA Technologies) were ligated. The ligation product was enriched with 15 PCR cycles and the final library was validated on an Agilent 2100 Bioanalyzer with the DNA 7500 assay. The libraries were sequenced on HiSeq 4000 (Illumina) with a read length of 2x51bp using the HiSeq 4000 SBS kit (Illumina). Primary data analysis, image analysis, base calling and quality scoring of the run were processed using the manufacturer’s software Real Time Analysis (RTA 2.7.7) followed by generation FASTQ sequence files.

**RNA-seq and pathway analysis**

RNA-seq reads were mapped against human reference genome (GRCh38) using STAR software version 2.5.3a[2]with ENCODE parameters. Annotated genes were quantified using human GENCODE annotation file version 34 with RSEM v1.3.0 [3]and default parameters. Differential expression analysis was performed with DESeq2 v1.26.0 R package [4]using a Wald test to compare affected and control groups, adjusting for sex in the model. Genes were considered differentially expressed with an adjusted p-value < 0.05 and absolute fold change |FC| > 1.5. A Gene Ontology enrichment analysis was generated with the significant genes using gProfileR v.07.0 [5]. Additionally, a GSEA was performed with a list of pre-ranked genes by the Wald statistic, using fgsea R package v1.12.0 [6]and the Reactome pathways database. PCA plot was generated with regularized log transformed (rlog) counts, considering only the top 500 most variable genes. Heatmap plot also represents rlog counts to show scaled expression of the top 50 differentially expressed genes. These plots were generated using ggplot2 v0.1.8 [7]and pheatmap R packages, respectively.

Moreover, pathway analysis was performed following the Core Analysis module of the IPA (QIAGEN Redwood City, www. qiagen.com/ingenuity) to identify canonical pathways, upstream regulators and top networks using a Fisher’s exact test with a cut-off of p-value adj <0.05. In addition, the Pathcards database [8] was used to relate DEGs to the pathways of interest.

**Autophagy protein array**

Cell lysates were obtained from IBM and CTL fibroblasts with cell lysis buffer containing 1% protease cocktail inhibitor (Thermo Scientific #78430, Massachusetts, USA). 250-500 ug of protein per sample were loaded in the RayBio® C Series Human Autophagy Array 1 (Cat#: AAHATG-1-8, RayBiotech, Inc., Atlanta, GA, USA) and incubated overnight at 4ºC in a pre-coated membrane, following the manufacturer’s protocol. The density of each spot pixel on the membrane was determined by chemoluminescent signaling, using the Image Quant TL Software (GE Healthcare), and calculated by AAH-ATG-1 analysis tools provided by RayBiotech, Inc. (Atlanta, USA).

**Autophagy time-course**

Autophagy flux in IBM vs. CTL fibroblasts was measured in a time-course manner in basal conditions (0h time point), and after the addition of 4 and 8h of 0.1 µM bafilomycin A1 from *Streptomyces griseus* (Sigma-Aldrich® #B1793 SIGMA, Missouri, USA), an inhibitor of the autophagic process aimed to block autophagosome clearance. Afterward, fibroblasts were lysed with RIPA buffer (Sigma-Aldrich #R0278, Missouri, USA) containing protease inhibitor cocktail (Thermo Scientific #78430, Massachusetts, USA) and analyzed through western blot analysis. Blots were probed against the anti- SQSTM1/p62 (Abcam #ab56416, Cambridge, UK) and anti-LC3B (Cell Signaling #2775S, Massachusets, USA) antibodies. LC3BII and p62 are considered autophagy markers and were normalized by total protein content (measured with SYPRO Ruby Protein Blot Stain, Molecular Probes). The intensity of signals was quantified by densitometric analysis of chemiluminescent signal (Image Quant TL Software, GE Healthcare).

**Immunocytochemistry for autophagosome characterization**

Cells were seeded in a 16-well glass slide (Nunc™ #178599 Lab-Tek® Chamber Slide™, Austin, USA) at 37ºC with 5% CO_2_ for 24 h. They were fixed with 4% paraformaldehyde for 15 min and permeabilized with 0.1% Triton X-100 in blocking solution (1% bovine serum albumin). Degradation of autophagolysosomes was blocked by adding 100nM Bafilomycin A1 from *Streptomyces griseus* (Sigma-Aldrich® #B1793 SIGMA, Missouri, USA) for 6 hours. Autophagosomes were stained by 1h incubation with anti-LC3 pAB (MBL International® #PM036, Massachusets, USA) and secondary marked through the donkey anti-rabbit Alexa Fluor® 488 IgG antibody (Life Technologies Europe, NL). Counterstain with DAPI was performed for nuclei staining (DAPI Fluoromount-G® #0100-20, Southern Biotech, Alabama, USA). Images were obtained with a Zeiss LSM 880 laser scanning confocal system using a 63X oil immersion objective.

**Mitochondrial Respiration**

Mitochondrial respiration was measured according to the oxygen consumption of the mitochondrial respiratory chain (MRC). The oxygen consumption rate was detected with the XF Cell Mito Stress Test™ (Seahorse-XF^e^24-Analyzer, Agilent Technologies), according to the manufacturer's protocol. Briefly, we seeded 40,000 fibroblasts per well in 24-well Seahorse culture plates and left them to adhere overnight. Each cell line was seeded in quadruplicate per condition. Basal oxygen consumption was measured before the addition of oligomycin (2µM, to measure MRC coupling), FCCP (1.4µM, uncoupling agent, to measure maximal oxygen consumption capacity) and antimycin A and rotenone addition (1µM each; to measure unspecific oxygen consumption of non-mitochondrial enzymes). Results were expressed relative to total protein (BCA) and mitochondrial content (CS concentration) as nmol/min/mg protein/CS.

**Oxidative stress**

Oxidative stress was measured through lipid peroxidation and total antioxidant capacity of the cell (TAC). Briefly, lipid peroxidation was quantified by measuring malondialdehyde (MDA) and 4-hydroxyalkenal (HAE) as indicators of ROS damage into cellular lipid compounds, using a BIOXYTECH® LPO-586™ colorimetric assay (Oxys International Inc., CA, USA), as reported elsewhere [9]. The results were normalized per mitochondrial content (µM MDA and HAE/mg protein/CS).

Concomitantly, TAC was quantified in cell culture supernatants using an OxiSelect™ Total Antioxidant Capacity Assay kit (Cell Biolabs Inc., San Diego, CA, USA) by spectrophotometry (absorption maximum at 490 nm), normalized by cell count and expressed as µM CRE (Copper Reducing Equivalents).

### **Transmission Electron Microscopy**

Fibroblasts were washed in PBS and fixed for 1 h in 2.5% glutaraldehyde in 0.1 M phosphate buffer at RT. Samples were gently scraped and pelleted in 1.5 ml tubes. Pellets were washed in PBS and incubated with 1% OsO_4_ for 90 min at 4 °C. Then samples were dehydrated, embedded in Spurr, and sectioned using Leica ultramicrotome (Leica Microsystems). Ultrathin sections (50–70 nm) were stained with 2% uranyl acetate for 10 min, a lead-staining solution for 5 min, and observed using a TEM, JEOL JEM-1010 fitted with a Gatan Orius SC1000 (model 832) digital camera [10] to seek for abnormal organelle structures.

**Metabolite quantification**

All fibroblasts' samples were quantified using a BCA protein assay (Thermo Scientific #23225, Massachusetts, USA) and 5 mg/ml were resuspended in 200 µL of PBS, and centrifuged (1500× g; 10 min) to collect the supernatant, where aa and organic acids were quantified. Organic acids were extracted in fibroblasts with ethyl acetate and diethyl ether and derivatized with bis(trimethylsilyl) trifluoro-acetamide, as previously reported [11]. The trimethylsilyl derivatives obtained were separated by gas chromatography (Agilent 7890A, Wilmington, DE, USA) and detected in a mass spectrometer (Agilent 5975C, Wilmington, DE, USA). The results were expressed as nanomoles of organic acid per milligram of protein (nmol/mg protein). Aa were quantified in fibroblasts by ultra-performance liquid chromatography coupled to tandem mass spectrometry, as previously reported [12]. Briefly, aa were separated in a Waters ACQUITY UPLC H-class chromatograph and quantified with a Waters Xevo TQD triple-quadrupole mass spectrometer using positive electrospray ionization conditions in the multiple reaction monitoring mode. The results were expressed as nanomoles of aa per milligram of protein (nmol/mg protein).

**Supplementary References (from Supplementary Methods section)**

1. MR R. 188th ENMC International Workshop: Inclusion Body Myositis, 2-4 December 2011, Naarden, The Netherlands. *Neuromuscul Disord*. 2013,23,1044–55.

2. Dobin A, Davis CA, Schlesinger F, Drenkow J, Zaleski C, Jha S, et al. STAR: ultrafast universal RNA-seq aligner. *Bioinformatics*. 2013,29,15–21.

3. Li B, Dewey CN. RSEM: accurate transcript quantification from RNA-Seq data with or without a reference genome. *BMC Bioinformatics 2011 12:1*. 2011,12,1–16.

4. Love MI, Huber W, Anders S. Moderated estimation of fold change and dispersion for RNA-seq data with DESeq2. *Genome Biology 2014 15:12*. 2014,15,1–21.

5. Reimand J, Kull M, Peterson H, Hansen J, Vilo J. g:Profiler—a web-based toolset for functional profiling of gene lists from large-scale experiments. *Nucleic Acids Research*. 2007,35,W193–200.

6. Korotkevich G, Sukhov V, Budin N, Shpak B, Artyomov M, Sergushichev A. Fast gene set enrichment analysis. 2016,.

7. Wickham H. ggplot2: Elegant Graphics for Data Analysis. New York: Springer-Verlag; 2009.

8. Belinky F, Nativ N, Stelzer G, Zimmerman S, Stein TI, Safran M, et al. PathCards: Multi-source consolidation of human biological pathways. *Database*. 2015,2015,1–13.

9. Catalán-García M, Garrabou G, Morén C, Guitart-Mampel M, Hernando A, Díaz-Ramos À, et al. Mitochondrial DNA disturbances and deregulated expression of oxidative phosphorylation and mitochondrial fusion proteins in sporadic inclusion body myositis. *Clinical Science*. 2016,130,1741–51.

10. Meneses-Salas E, García-Melero A, Kanerva K, Blanco-Muñoz P, Morales-Paytuvi F, Bonjoch J, et al. Annexin A6 modulates TBC1D15/Rab7/StARD3 axis to control endosomal cholesterol export in NPC1 cells. *Cellular and Molecular Life Sciences*. 2020,77,2839–57.

11. Hoffmann GF, Feyh P. Organic Acid Analysis. In: Physician’s Guide to the Laboratory Diagnosis of Metabolic Diseases. Berlin, Heidelberg: Springer Berlin Heidelberg; 2003. p. 27–44.

12. Casado M, Sierra C, Batllori M, Artuch R, Ormazabal A. A targeted metabolomic procedure for amino acid analysis in different biological specimens by ultra-high-performance liquid chromatography–tandem mass spectrometry. *Metabolomics*. 2018,14,1–12.

**SUPPLEMENTARY TABLES**

**Supplementary Table 1**. Differentially expressed genes between IBM and CTL (778 genes with p-value adj (FDR) < 0.05, following alphabetical order)

| **GENE ID** | **GENE NAME** | **log2Fold change** | **p-value** | **p-value adj (FDR)** |
| --- | --- | --- | --- | --- |
| ABCC9 | ATP binding cassette subfamily C member 9(ABCC9) | 5.2418 | 0.0000 | 0.0001 |
| ABHD12 | abhydrolase domain containing 12(ABHD12) | -0.6465 | 0.0000 | 0.0000 |
| ABI3BP | ABI family member 3 binding protein (ABI3BP) | -1.4671 | 0.0002 | 0.0092 |
| ABLIM3 | actin binding LIM protein family member 3(ABLIM3) | -0.8941 | 0.0003 | 0.0118 |
| AC004556.3 | unknown | 24.8812 | 0.0000 | 0.0000 |
| AC007405.4 | unknown | 3.7031 | 0.0007 | 0.0209 |
| AC008764.4 | unknown | 6.7625 | 0.0000 | 0.0005 |
| AC009779.4 | unknown | -1.5096 | 0.0001 | 0.0052 |
| AC010186.2 | unknown | 1.0420 | 0.0021 | 0.0464 |
| AC011005.1 | unknown | -2.9930 | 0.0022 | 0.0484 |
| AC011484.1 | unknown | 1.5290 | 0.0009 | 0.0259 |
| AC012531.1 | unknown | 3.4745 | 0.0018 | 0.0418 |
| AC012651.1 | unknown | -1.1590 | 0.0002 | 0.0091 |
| AC016026.1 | unknown | -0.8236 | 0.0001 | 0.0034 |
| AC023055.1 | unknown | -4.5706 | 0.0002 | 0.0085 |
| AC027097.2 | unknown | 2.0449 | 0.0005 | 0.0159 |
| AC034102.2 | unknown | 2.5011 | 0.0009 | 0.0247 |
| AC067968.1 | unknown | 6.6426 | 0.0010 | 0.0270 |
| AC078850.1 | unknown | 2.3634 | 0.0008 | 0.0229 |
| AC104452.1 | unknown | -0.8546 | 0.0002 | 0.0079 |
| AC104461.1 | unknown | 4.9800 | 0.0004 | 0.0135 |
| AC106782.1 | unknown | -1.8723 | 0.0001 | 0.0063 |
| AC106795.2 | unknown | -2.7117 | 0.0001 | 0.0029 |
| AC110079.1 | unknown | 1.5728 | 0.0002 | 0.0070 |
| AC138894.1 | unknown | -1.5118 | 0.0005 | 0.0168 |
| AC138969.3 | unknown | 4.2973 | 0.0010 | 0.0274 |
| AC243964.4 | unknown | -1.5044 | 0.0004 | 0.0143 |
| ACADM | acyl-CoA dehydrogenase, C-4 to C-12 straight chain (ACADM) | 0.5216 | 0.0015 | 0.0369 |
| ACAN | aggrecan (ACAN) | 7.0350 | 0.0000 | 0.0002 |
| ACKR4 | atypical chemokine receptor 4(ACKR4) | -2.1493 | 0.0001 | 0.0029 |
| ACSL5 | acyl-CoA synthetase long-chain family member 5(ACSL5) | 2.3992 | 0.0002 | 0.0068 |
| ACTC1 | actin, alpha, cardiac muscle 1(ACTC1) | 2.3333 | 0.0000 | 0.0018 |
| ADAM15 | ADAM metallopeptidase domain 15(ADAM15) | -0.8433 | 0.0002 | 0.0078 |
| ADAMTS14 | ADAM metallopeptidase with thrombospondin type 1 motif 14(ADAMTS14) | -1.2174 | 0.0000 | 0.0009 |
| ADAMTS8 | ADAM metallopeptidase with thrombospondin type 1 motif 8(ADAMTS8) | -2.4147 | 0.0005 | 0.0172 |
| ADAMTSL4 | ADAMTS like 4(ADAMTSL4) | -1.7184 | 0.0000 | 0.0001 |
| ADAMTSL5 | ADAMTS like 5(ADAMTSL5) | -0.9505 | 0.0000 | 0.0012 |
| ADGRE5 | adhesion G protein-coupled receptor E5(ADGRE5) | -0.7142 | 0.0002 | 0.0089 |
| ADIRF | adipogenesis regulatory factor (ADIRF) | -1.1283 | 0.0006 | 0.0184 |
| ADM2 | adrenomedullin 2(ADM2) | 0.7624 | 0.0000 | 0.0015 |
| ADRA1D | adrenoceptor alpha 1D(ADRA1D) | -2.1892 | 0.0000 | 0.0000 |
| ADRA2A | adrenoceptor alpha 2A(ADRA2A) | -1.8182 | 0.0005 | 0.0171 |
| AFAP1 | actin filament associated protein 1(AFAP1) | -0.8843 | 0.0012 | 0.0304 |
| AGAP7P | ArfGAP with GTPase domain, ankyrin repeat and PH domain 7, pseudogene (AGAP7P) | -4.8467 | 0.0000 | 0.0010 |
| AGL | amylo-alpha-1, 6-glucosidase, 4-alpha-glucanotransferase (AGL) | 0.5079 | 0.0004 | 0.0132 |
| AGPAT2 | 1-acylglycerol-3-phosphate O-acyltransferase 2(AGPAT2) | -0.4554 | 0.0002 | 0.0095 |
| AGPS | alkylglycerone phosphate synthase (AGPS) | 0.4610 | 0.0001 | 0.0031 |
| AHNAK2 | AHNAK nucleoprotein 2(AHNAK2) | -1.4524 | 0.0000 | 0.0000 |
| AHSA2P | Activator Of HSP90 ATPase Homolog 2, Pseudogene | -0.5280 | 0.0011 | 0.0278 |
| AKNA | AT-hook transcription factor (AKNA) | 1.0736 | 0.0000 | 0.0001 |
| AL049629.2 | unknown | -1.0896 | 0.0000 | 0.0000 |
| AL354740.1 | unknown | -1.5696 | 0.0000 | 0.0024 |
| AL365203.2 | unknown | -0.9654 | 0.0011 | 0.0289 |
| AL365205.1 | unknown | -1.1985 | 0.0000 | 0.0001 |
| AL627309.6 | unknown | -2.3974 | 0.0001 | 0.0050 |
| AL928654.3 | unknown | -1.7488 | 0.0022 | 0.0480 |
| ALDH18A1 | aldehyde dehydrogenase 18 family member A1(ALDH18A1) | 0.3572 | 0.0023 | 0.0498 |
| ALDH1B1 | aldehyde dehydrogenase 1 family member B1(ALDH1B1) | 1.2248 | 0.0000 | 0.0011 |
| ALDH1L2 | aldehyde dehydrogenase 1 family member L2(ALDH1L2) | 0.6341 | 0.0000 | 0.0018 |
| ALDH3A1 | aldehyde dehydrogenase 3 family member A1(ALDH3A1) | -2.5892 | 0.0001 | 0.0063 |
| ALDH4A1 | aldehyde dehydrogenase 4 family member A1(ALDH4A1) | -0.8059 | 0.0003 | 0.0104 |
| ANK1 | ankyrin 1(ANK1) | -3.0735 | 0.0000 | 0.0006 |
| ANKRD13A | ankyrin repeat domain 13A(ANKRD13A) | -0.4556 | 0.0000 | 0.0012 |
| ANKRD44 | ankyrin repeat domain 44(ANKRD44) | 0.8013 | 0.0009 | 0.0248 |
| ANKRD50 | ankyrin repeat domain 50(ANKRD50) | 0.5588 | 0.0003 | 0.0117 |
| ANKRD6 | ankyrin repeat domain 6(ANKRD6) | 2.5131 | 0.0000 | 0.0003 |
| ANO4 | anoctamin 4(ANO4) | -3.3334 | 0.0000 | 0.0000 |
| ANPEP | alanyl aminopeptidase, membrane (ANPEP) | -0.6780 | 0.0000 | 0.0002 |
| AP001273.2 | unknown | 7.6508 | 0.0000 | 0.0020 |
| AP003071.4 | unknown | -1.2800 | 0.0009 | 0.0254 |
| APBB1 | amyloid beta precursor protein binding family B member 1(APBB1) | -0.5300 | 0.0000 | 0.0010 |
| APCDD1L | APC downregulated 1 like (APCDD1L) | -0.8223 | 0.0012 | 0.0296 |
| APH1B | aph-1 homolog B, gamma-secretase subunit (APH1B) | -0.5951 | 0.0001 | 0.0029 |
| APMAP | adipocyte plasma membrane associated protein (APMAP) | -0.6084 | 0.0015 | 0.0356 |
| APOL1 | apolipoprotein L1(APOL1) | -0.8892 | 0.0003 | 0.0112 |
| AR | androgen receptor (AR) | 3.7771 | 0.0000 | 0.0001 |
| ARG2 | arginase 2(ARG2) | 0.9386 | 0.0002 | 0.0080 |
| ARHGAP20 | Rho GTPase activating protein 20(ARHGAP20) | 1.9725 | 0.0008 | 0.0220 |
| ARHGAP23 | Rho GTPase activating protein 23(ARHGAP23) | -0.6173 | 0.0002 | 0.0092 |
| ARHGAP27P1-BPTFP1-KPNA2P3 | ARHGAP27P1-BPTFP1-KPNA2P3 Readthrough, Transcribed Pseudogene | -0.7563 | 0.0000 | 0.0004 |
| ARHGEF2 | Rho/Rac guanine nucleotide exchange factor 2(ARHGEF2) | 0.4460 | 0.0010 | 0.0270 |
| ARHGEF28 | Rho guanine nucleotide exchange factor 28(ARHGEF28) | -1.0330 | 0.0018 | 0.0407 |
| ARHGEF5 | Rho guanine nucleotide exchange factor 5(ARHGEF5) | 0.8280 | 0.0022 | 0.0484 |
| ARID5A | AT-rich interaction domain 5A(ARID5A) | -1.1437 | 0.0004 | 0.0126 |
| ARMH4 | Armadillo Like Helical Domain Containing 4 | -0.8213 | 0.0008 | 0.0221 |
| ARMT1 | acidic residue methyltransferase 1(ARMT1) | 0.5181 | 0.0010 | 0.0275 |
| ARPC1A | actin related protein 2/3 complex subunit 1A(ARPC1A) | -0.3344 | 0.0018 | 0.0417 |
| ARRDC1 | arrestin domain containing 1(ARRDC1) | -0.6306 | 0.0000 | 0.0007 |
| ARSD | arylsulfatase D(ARSD) | -0.6414 | 0.0001 | 0.0046 |
| ASNS | asparagine synthetase (glutamine-hydrolyzing) (ASNS) | 1.1373 | 0.0000 | 0.0000 |
| ATP13A3 | ATPase 13A3(ATP13A3) | -0.6369 | 0.0001 | 0.0044 |
| ATP1B3 | ATPase Na+/K+ transporting subunit beta 3(ATP1B3) | -0.5233 | 0.0011 | 0.0282 |
| ATP2B4 | ATPase plasma membrane Ca2+ transporting 4(ATP2B4) | -1.0839 | 0.0000 | 0.0000 |
| ATP2C1 | ATPase secretory pathway Ca2+ transporting 1(ATP2C1) | -0.3462 | 0.0014 | 0.0355 |
| ATP6V0A1 | ATPase H+ transporting V0 subunit a1(ATP6V0A1) | -0.7267 | 0.0006 | 0.0191 |
| ATP6V0E2 | ATPase H+ transporting V0 subunit e2(ATP6V0E2) | -0.8363 | 0.0008 | 0.0241 |
| ATP6V1G1 | ATPase H+ transporting V1 subunit G1(ATP6V1G1) | 0.5128 | 0.0000 | 0.0026 |
| ATP8B1 | ATPase phospholipid transporting 8B1(ATP8B1) | -1.2718 | 0.0000 | 0.0000 |
| AUP1 | ancient ubiquitous protein 1(AUP1) | -0.3966 | 0.0003 | 0.0104 |
| B4GAT1 | beta-1,4-glucuronyltransferase 1(B4GAT1) | -0.6639 | 0.0017 | 0.0403 |
| BAIAP2 | BAI1 associated protein 2(BAIAP2) | -0.6732 | 0.0010 | 0.0264 |
| BANK1 | B-cell scaffold protein with ankyrin repeats 1(BANK1) | 2.9500 | 0.0000 | 0.0018 |
| BCL11A | B-cell CLL/lymphoma 11A(BCL11A) | 1.9714 | 0.0003 | 0.0114 |
| BCL2L1 | BCL2 like 1(BCL2L1) | -0.6640 | 0.0000 | 0.0000 |
| BDKRB1 | bradykinin receptor B1(BDKRB1) | -1.4603 | 0.0000 | 0.0001 |
| BSCL2 | BSCL2, seipin lipid droplet biogenesis associated (BSCL2) | -0.7716 | 0.0000 | 0.0002 |
| BSG | basigin (Ok blood group) (BSG) | -0.6915 | 0.0002 | 0.0081 |
| BTBD3 | BTB domain containing 3(BTBD3) | -0.5217 | 0.0019 | 0.0438 |
| BTF3L4 | basic transcription factor 3 like 4(BTF3L4) | 0.4380 | 0.0006 | 0.0195 |
| BTF3L4P2 | basic transcription factor 3 like 4 pseudogene 2(BTF3L4P2) | 1.1680 | 0.0004 | 0.0126 |
| BTN3A2 | butyrophilin subfamily 3 member A2(BTN3A2) | -1.0128 | 0.0016 | 0.0378 |
| BVES | blood vessel epicardial substance (BVES) | 0.6310 | 0.0010 | 0.0271 |
| C10orf105 | chromosome 10 open reading frame 105(C10orf105) | -1.8647 | 0.0001 | 0.0063 |
| C17orf107 | chromosome 17 open reading frame 107(C17orf107) | 0.9326 | 0.0020 | 0.0445 |
| C17orf97 | chromosome 17 open reading frame 97(C17orf97) | -1.4688 | 0.0000 | 0.0004 |
| C1QTNF2 | C1q and tumor necrosis factor related protein 2(C1QTNF2) | -1.3620 | 0.0019 | 0.0429 |
| C2orf74 | chromosome 2 open reading frame 74(C2orf74) | 1.3086 | 0.0000 | 0.0027 |
| C3orf18 | chromosome 3 open reading frame 18(C3orf18) | -0.6791 | 0.0000 | 0.0000 |
| C9orf64 | chromosome 9 open reading frame 64(C9orf64) | 0.6727 | 0.0000 | 0.0006 |
| CA13 | carbonic anhydrase 13(CA13) | 1.6189 | 0.0012 | 0.0307 |
| CADM3 | cell adhesion molecule 3(CADM3) | 5.0916 | 0.0000 | 0.0000 |
| CADPS2 | calcium dependent secretion activator 2(CADPS2) | 1.8008 | 0.0012 | 0.0313 |
| CAMKK1 | calcium/calmodulin dependent protein kinase kinase 1 (CAMKK1) | -0.5466 | 0.0017 | 0.0405 |
| CAPN3 | calpain 3(CAPN3) | -0.8838 | 0.0010 | 0.0274 |
| CARD10 | caspase recruitment domain family member 10(CARD10) | -1.4910 | 0.0001 | 0.0052 |
| CASC15 | cancer susceptibility candidate 15 (non-protein coding) (CASC15) | 3.1988 | 0.0001 | 0.0055 |
| CASP4 | caspase 4(CASP4) | 0.3903 | 0.0008 | 0.0229 |
| CATSPER1 | cation channel sperm associated 1(CATSPER1) | -2.4289 | 0.0009 | 0.0249 |
| CAV1 | caveolin 1(CAV1) | -1.1562 | 0.0000 | 0.0008 |
| CBR3 | carbonyl reductase 3(CBR3) | -0.7673 | 0.0000 | 0.0007 |
| CBWD4P | COBW domain containing 4 pseudogene (CBWD4P) | 3.6966 | 0.0010 | 0.0274 |
| CBX4 | chromobox 4(CBX4) | 0.6131 | 0.0004 | 0.0141 |
| CCDC106 | coiled-coil domain containing 106(CCDC106) | -0.4520 | 0.0009 | 0.0259 |
| CCDC144A | coiled-coil domain containing 144A(CCDC144A) | 7.7440 | 0.0000 | 0.0000 |
| CCDC170 | coiled-coil domain containing 170(CCDC170) | 1.5433 | 0.0002 | 0.0092 |
| CCDC84 | coiled-coil domain containing 84(CCDC84) | -0.6570 | 0.0000 | 0.0009 |
| CCN4 | Cellular Communication Network Factor 4 | 1.3436 | 0.0000 | 0.0000 |
| CCND1 | cyclin D1(CCND1) | -1.5007 | 0.0000 | 0.0000 |
| CCT2 | chaperonin containing TCP1 subunit 2(CCT2) | 0.3052 | 0.0010 | 0.0271 |
| CD151 | CD151 molecule (Raph blood group) (CD151) | -1.0611 | 0.0000 | 0.0006 |
| CD163 | CD163 molecule (CD163) | 2.2650 | 0.0019 | 0.0437 |
| CD248 | CD248 molecule (CD248) | -0.5876 | 0.0010 | 0.0271 |
| CD3EAP | CD3e molecule associated protein (CD3EAP) | 0.9539 | 0.0007 | 0.0209 |
| CD44 | CD44 molecule (Indian blood group) (CD44) | -0.8714 | 0.0003 | 0.0104 |
| CD47 | CD47 molecule (CD47) | -0.5105 | 0.0000 | 0.0006 |
| CD59 | CD59 molecule (CD59) | -0.8524 | 0.0000 | 0.0000 |
| CD63 | CD63 molecule (CD63) | -0.8199 | 0.0000 | 0.0003 |
| CD68 | CD68 molecule (CD68) | -1.1347 | 0.0000 | 0.0018 |
| CD81 | CD81 molecule (CD81) | -0.6728 | 0.0000 | 0.0014 |
| CD82 | CD82 molecule (CD82) | -1.6099 | 0.0002 | 0.0067 |
| CD9 | CD9 molecule (CD9) | -2.3911 | 0.0000 | 0.0000 |
| CD99 | CD99 molecule (CD99) | -0.4673 | 0.0000 | 0.0002 |
| CDC42EP2 | CDC42 effector protein 2(CDC42EP2) | -0.8919 | 0.0004 | 0.0126 |
| CDH18 | cadherin 18(CDH18) | -5.0507 | 0.0000 | 0.0003 |
| CDH6 | cadherin 6(CDH6) | 2.7957 | 0.0000 | 0.0001 |
| CDHR3 | cadherin related family member 3(CDHR3) | -1.0315 | 0.0001 | 0.0051 |
| CDRT4 | CMT1A duplicated region transcript 4(CDRT4) | -1.8932 | 0.0000 | 0.0001 |
| CEBPG | CCAAT/enhancer binding protein gamma (CEBPG) | 0.4814 | 0.0000 | 0.0018 |
| CELSR1 | cadherin EGF LAG seven-pass G-type receptor 1(CELSR1) | -2.9541 | 0.0003 | 0.0113 |
| CERCAM | cerebral endothelial cell adhesion molecule (CERCAM) | -0.7539 | 0.0013 | 0.0335 |
| CERS1 | ceramide synthase 1(CERS1) | -1.2438 | 0.0005 | 0.0158 |
| CFD | complement factor D(CFD) | 1.6744 | 0.0000 | 0.0000 |
| CHAC1 | ChaC glutathione specific gamma-glutamylcyclotransferase 1(CHAC1) | 1.7225 | 0.0003 | 0.0101 |
| CHDH | choline dehydrogenase (CHDH) | -2.1142 | 0.0001 | 0.0062 |
| CHN2 | chimerin 2(CHN2) | 5.1535 | 0.0001 | 0.0043 |
| CHPF | chondroitin polymerizing factor (CHPF) | -0.9390 | 0.0017 | 0.0404 |
| CHPT1 | choline phosphotransferase 1(CHPT1) | -0.3680 | 0.0010 | 0.0262 |
| CHST6 | carbohydrate sulfotransferase 6(CHST6) | 2.4710 | 0.0013 | 0.0321 |
| CITED2 | Cbp/p300 interacting transactivator with Glu/Asp rich carboxy-terminal domain 2(CITED2) | -0.5678 | 0.0001 | 0.0062 |
| CKB | creatine kinase B(CKB) | -1.6437 | 0.0000 | 0.0026 |
| CLDN23 | claudin 23(CLDN23) | 1.2082 | 0.0017 | 0.0389 |
| CLEC11A | C-type lectin domain family 11 member A(CLEC11A) | -0.9878 | 0.0000 | 0.0004 |
| CLEC12A | C-type lectin domain family 12 member A(CLEC12A) | -4.2622 | 0.0000 | 0.0000 |
| CLEC2A | C-type lectin domain family 2 member A(CLEC2A) | -7.3567 | 0.0000 | 0.0000 |
| CLEC2B | C-type lectin domain family 2 member B(CLEC2B) | -1.7995 | 0.0000 | 0.0000 |
| CLEC3B | C-type lectin domain family 3 member B(CLEC3B) | -3.2430 | 0.0000 | 0.0000 |
| CLGN | calmegin (CLGN) | 1.3861 | 0.0005 | 0.0168 |
| CLIC2 | chloride intracellular channel 2(CLIC2) | 3.8043 | 0.0000 | 0.0002 |
| CLIC6 | chloride intracellular channel 6(CLIC6) | 3.7952 | 0.0002 | 0.0065 |
| CLMN | calmin (CLMN) | 3.5275 | 0.0001 | 0.0062 |
| CLTB | clathrin light chain B(CLTB) | -0.5051 | 0.0000 | 0.0013 |
| CNN3 | calponin 3(CNN3) | 0.4807 | 0.0015 | 0.0369 |
| COL10A1 | collagen type X alpha 1 chain (COL10A1) | 2.2962 | 0.0019 | 0.0432 |
| COL13A1 | collagen type XIII alpha 1 chain (COL13A1) | -1.1746 | 0.0000 | 0.0003 |
| COL4A4 | collagen type IV alpha 4 chain (COL4A4) | 5.2061 | 0.0000 | 0.0024 |
| COPB2 | coatomer protein complex subunit beta 2(COPB2) | 0.3450 | 0.0019 | 0.0432 |
| COPZ2 | coatomer protein complex subunit zeta 2(COPZ2) | -0.3562 | 0.0006 | 0.0191 |
| CPB1 | carboxypeptidase B1(CPB1) | 4.0747 | 0.0008 | 0.0241 |
| CPED1 | cadherin like and PC-esterase domain containing 1(CPED1) | -1.2292 | 0.0010 | 0.0267 |
| CPM | carboxypeptidase M(CPM) | 3.3745 | 0.0000 | 0.0000 |
| CRELD1 | cysteine rich with EGF like domains 1(CRELD1) | -1.2557 | 0.0010 | 0.0267 |
| CRIP1 | cysteine rich protein 1(CRIP1) | -1.7549 | 0.0000 | 0.0000 |
| CRIP2 | cysteine rich protein 2(CRIP2) | -0.6618 | 0.0001 | 0.0034 |
| CRTAP | cartilage associated protein (CRTAP) | -0.5255 | 0.0018 | 0.0417 |
| CRYAB | crystallin alpha B(CRYAB) | -0.5729 | 0.0009 | 0.0242 |
| CRYBG1 | Crystallin Beta-Gamma Domain Containing 1 | -1.4347 | 0.0000 | 0.0018 |
| CST3 | cystatin C(CST3) | -0.9566 | 0.0002 | 0.0073 |
| CST6 | cystatin E/M(CST6) | -2.5056 | 0.0001 | 0.0035 |
| CSTF2T | cleavage stimulation factor subunit 2 tau variant (CSTF2T) | 0.4714 | 0.0021 | 0.0461 |
| CTDSPL | CTD small phosphatase like (CTDSPL) | -0.4733 | 0.0011 | 0.0282 |
| CTH | cystathionine gamma-lyase (CTH) | 0.9054 | 0.0007 | 0.0211 |
| CTSB | cathepsin B(CTSB) | -0.7951 | 0.0000 | 0.0010 |
| CTSC | cathepsin C(CTSC) | -1.9871 | 0.0000 | 0.0000 |
| CTSZ | cathepsin Z(CTSZ) | -0.8578 | 0.0013 | 0.0318 |
| CU633904.2 | unknown | 3.6259 | 0.0000 | 0.0000 |
| CU634019.2 | unknown | 4.0153 | 0.0000 | 0.0005 |
| CXCL14 | C-X-C motif chemokine ligand 14(CXCL14) | 2.2072 | 0.0018 | 0.0406 |
| CYB5A | cytochrome b5 type A(CYB5A) | 0.6328 | 0.0015 | 0.0360 |
| CYB5D2 | cytochrome b5 domain containing 2(CYB5D2) | -0.5325 | 0.0006 | 0.0178 |
| CYB5R3 | cytochrome b5 reductase 3(CYB5R3) | -0.5536 | 0.0021 | 0.0464 |
| CYP2U1 | cytochrome P450 family 2 subfamily U member 1(CYP2U1) | -1.1982 | 0.0000 | 0.0000 |
| CYP4V2 | cytochrome P450 family 4 subfamily V member 2(CYP4V2) | -0.6433 | 0.0007 | 0.0207 |
| CYP7B1 | cytochrome P450 family 7 subfamily B member 1(CYP7B1) | 2.9934 | 0.0000 | 0.0000 |
| DAB2 | DAB2, clathrin adaptor protein (DAB2) | 0.3330 | 0.0003 | 0.0104 |
| DACT1 | dishevelled binding antagonist of beta catenin 1(DACT1) | 2.7342 | 0.0000 | 0.0000 |
| DAG1 | dystroglycan 1(DAG1) | -0.6364 | 0.0014 | 0.0352 |
| DARS1 | Aspartyl-TRNA Synthetase 1 | 0.4035 | 0.0004 | 0.0126 |
| DDIT3 | DNA damage inducible transcript 3(DDIT3) | 0.4761 | 0.0001 | 0.0052 |
| DDIT4 | DNA damage inducible transcript 4(DDIT4) | 1.3773 | 0.0001 | 0.0029 |
| DDX18 | DEAD-box helicase 18(DDX18) | 0.4255 | 0.0002 | 0.0070 |
| DENND3 | DENN domain containing 3(DENND3) | -1.3941 | 0.0000 | 0.0000 |
| DEPP1 | DEPP1 Autophagy Regulator | 2.0641 | 0.0001 | 0.0049 |
| DEPTOR | DEP domain containing MTOR-interacting protein (DEPTOR) | 1.3954 | 0.0000 | 0.0002 |
| DGCR11 | DiGeorge syndrome critical region gene 11 (non-protein coding) (DGCR11) | -1.2104 | 0.0002 | 0.0067 |
| DGCR2 | DiGeorge syndrome critical region gene 2(DGCR2) | -0.4936 | 0.0006 | 0.0175 |
| DIAPH1 | diaphanous related formin 1(DIAPH1) | -0.4682 | 0.0004 | 0.0149 |
| DIO2 | deiodinase, iodothyronine type II(DIO2) | 2.8134 | 0.0001 | 0.0039 |
| DLEU2 | deleted in lymphocytic leukemia 2 (non-protein coding) (DLEU2) | 0.8991 | 0.0011 | 0.0278 |
| DMKN | dermokine (DMKN) | 2.2967 | 0.0000 | 0.0008 |
| DMPK | dystrophia myotonica protein kinase (DMPK) | -0.8460 | 0.0000 | 0.0000 |
| DNM3OS | DNM3 opposite strand/antisense RNA(DNM3OS) | 0.9808 | 0.0008 | 0.0229 |
| DPP7 | dipeptidyl peptidase 7(DPP7) | -0.5894 | 0.0004 | 0.0138 |
| DPT | dermatopontin (DPT) | 1.3498 | 0.0002 | 0.0093 |
| DSE | dermatan sulfate epimerase (DSE) | 0.6231 | 0.0005 | 0.0157 |
| DUOX1 | dual oxidase 1(DUOX1) | -3.4372 | 0.0010 | 0.0264 |
| ECHDC2 | enoyl-CoA hydratase domain containing 2(ECHDC2) | -0.7406 | 0.0004 | 0.0126 |
| EDNRB | endothelin receptor type B(EDNRB) | 6.8732 | 0.0000 | 0.0008 |
| EGFL6 | EGF like domain multiple 6(EGFL6) | 7.0166 | 0.0012 | 0.0314 |
| EGFR | epidermal growth factor receptor (EGFR) | -0.5891 | 0.0006 | 0.0190 |
| EHD1 | EH domain containing 1(EHD1) | -0.9371 | 0.0000 | 0.0000 |
| EHD3 | EH domain containing 3(EHD3) | -0.9221 | 0.0013 | 0.0319 |
| EIF4EBP1 | eukaryotic translation initiation factor 4E binding protein 1(EIF4EBP1) | 0.7723 | 0.0000 | 0.0018 |
| ELFN1 | extracellular leucine rich repeat and fibronectin type III domain containing 1(ELFN1) | -1.4073 | 0.0004 | 0.0141 |
| EMCN | endomucin (EMCN) | 2.9971 | 0.0010 | 0.0262 |
| EMILIN2 | elastin microfibril interfacer 2(EMILIN2) | -0.9512 | 0.0000 | 0.0018 |
| EML5 | echinoderm microtubule associated protein like 5(EML5) | 5.8734 | 0.0000 | 0.0001 |
| EMP1 | epithelial membrane protein 1(EMP1) | -0.6413 | 0.0010 | 0.0262 |
| EMP3 | epithelial membrane protein 3(EMP3) | -0.6530 | 0.0000 | 0.0000 |
| ENAH | enabled homolog (Drosophila)(ENAH) | 0.3909 | 0.0000 | 0.0021 |
| EPDR1 | ependymin related 1(EPDR1) | -2.5181 | 0.0000 | 0.0000 |
| EPRS1 | Glutamyl-Prolyl-TRNA Synthetase 1 | 0.4312 | 0.0000 | 0.0009 |
| ERGIC1 | endoplasmic reticulum-golgi intermediate compartment 1(ERGIC1) | -0.5988 | 0.0001 | 0.0029 |
| ERGIC2 | ERGIC and golgi 2(ERGIC2) | -0.3696 | 0.0010 | 0.0274 |
| ERICH2 | glutamate rich 2(ERICH2) | 4.9875 | 0.0013 | 0.0327 |
| ERV3-1 | endogenous retrovirus group 3 member 1(ERV3-1) | 1.3180 | 0.0000 | 0.0000 |
| ETHE1 | ETHE1, persulfide dioxygenase (ETHE1) | -1.2257 | 0.0000 | 0.0007 |
| ETS2 | ETS proto-oncogene 2, transcription factor (ETS2) | -0.8156 | 0.0000 | 0.0000 |
| EXOSC6 | exosome component 6(EXOSC6) | 0.5235 | 0.0001 | 0.0054 |
| EXOSC8 | exosome component 8(EXOSC8) | 0.5764 | 0.0014 | 0.0344 |
| EXOSC9 | exosome component 9(EXOSC9) | 0.6994 | 0.0001 | 0.0061 |
| F11R | F11 receptor(F11R) | 3.8174 | 0.0015 | 0.0363 |
| FAM156B | family with sequence similarity 156 member B(FAM156B) | -0.8472 | 0.0017 | 0.0396 |
| FAM174B | family with sequence similarity 174 member B(FAM174B) | 2.1618 | 0.0004 | 0.0126 |
| FAM180A | family with sequence similarity 180 member A(FAM180A) | -1.2671 | 0.0000 | 0.0000 |
| FAM20C | FAM20C, golgi associated secretory pathway kinase (FAM20C) | -0.4772 | 0.0010 | 0.0262 |
| FAM210B | family with sequence similarity 210 member B(FAM210B) | -0.5666 | 0.0000 | 0.0020 |
| FAM66B | family with sequence similarity 66 member B(FAM66B) | 1.5810 | 0.0000 | 0.0000 |
| FAR2 | fatty acyl-CoA reductase 2(FAR2) | 3.0102 | 0.0000 | 0.0000 |
| FARP1 | FERM, ARH/RhoGEF and pleckstrin domain protein 1(FARP1) | -0.5316 | 0.0021 | 0.0459 |
| FBXW5 | F-box and WD repeat domain containing 5(FBXW5) | -0.3916 | 0.0000 | 0.0016 |
| FCRLB | Fc receptor like B(FCRLB) | -1.2278 | 0.0007 | 0.0211 |
| FITM2 | fat storage inducing transmembrane protein 2(FITM2) | -0.5422 | 0.0004 | 0.0135 |
| FKBP8 | FK506 binding protein 8(FKBP8) | -0.2935 | 0.0012 | 0.0301 |
| FLOT2 | flotillin 2(FLOT2) | -0.5627 | 0.0000 | 0.0002 |
| FMN1 | formin 1(FMN1) | 1.7393 | 0.0000 | 0.0001 |
| FN3K | fructosamine 3 kinase (FN3K) | -0.8439 | 0.0003 | 0.0114 |
| FNDC1 | fibronectin type III domain containing 1(FNDC1) | 2.6758 | 0.0000 | 0.0000 |
| FNDC4 | fibronectin type III domain containing 4(FNDC4) | -0.9180 | 0.0000 | 0.0017 |
| FOLR3 | folate receptor 3(FOLR3) | -2.3511 | 0.0000 | 0.0003 |
| FOSL1 | FOS like 1, AP-1 transcription factor subunit (FOSL1) | -0.4624 | 0.0000 | 0.0000 |
| FOXC2 | forkhead box C2(FOXC2) | -2.3388 | 0.0017 | 0.0393 |
| FOXD1 | forkhead box D1(FOXD1) | 0.6955 | 0.0002 | 0.0085 |
| FOXP1 | forkhead box P1(FOXP1) | 0.6402 | 0.0000 | 0.0002 |
| FOXP4 | forkhead box P4(FOXP4) | 0.4798 | 0.0001 | 0.0054 |
| FOXQ1 | forkhead box Q1(FOXQ1) | -2.3371 | 0.0000 | 0.0005 |
| FRMD8 | FERM domain containing 8(FRMD8) | -0.5383 | 0.0018 | 0.0417 |
| FRRS1 | ferric chelate reductase 1(FRRS1) | 1.6726 | 0.0002 | 0.0075 |
| FST | follistatin (FST) | -1.1043 | 0.0000 | 0.0000 |
| FUCA2 | fucosidase, alpha-L- 2, plasma (FUCA2) | -0.8001 | 0.0000 | 0.0002 |
| FZD1 | frizzled class receptor 1(FZD1) | 0.6714 | 0.0000 | 0.0006 |
| GAB1 | GRB2 associated binding protein 1(GAB1) | 0.6600 | 0.0021 | 0.0464 |
| GABRE | gamma-aminobutyric acid type A receptor epsilon subunit (GABRE) | 0.8693 | 0.0000 | 0.0007 |
| GALNT16 | polypeptide N-acetylgalactosaminyltransferase 16(GALNT16) | -2.0373 | 0.0002 | 0.0077 |
| GAMT | guanidinoacetate N-methyltransferase (GAMT) | -0.6399 | 0.0021 | 0.0457 |
| GARS1 | Glycyl-TRNA Synthetase 1 | 0.5152 | 0.0000 | 0.0000 |
| GART | phosphoribosylglycinamide formyltransferase, phosphoribosylglycinamide synthetase, phosphoribosylaminoimidazole synthetase (GART) | 0.5124 | 0.0000 | 0.0001 |
| GAS5 | growth arrest specific 5 (non-protein coding) (GAS5) | 0.4187 | 0.0000 | 0.0021 |
| GAS7 | growth arrest specific 7(GAS7) | 1.4805 | 0.0000 | 0.0004 |
| GCNT1 | glucosaminyl (N-acetyl) transferase 1, core 2(GCNT1) | -1.0822 | 0.0000 | 0.0004 |
| GDF10 | growth differentiation factor 10(GDF10) | 4.2503 | 0.0000 | 0.0005 |
| GDF5 | growth differentiation factor 5(GDF5) | -2.3219 | 0.0004 | 0.0145 |
| GLIPR1 | GLI pathogenesis related 1(GLIPR1) | -1.0883 | 0.0014 | 0.0351 |
| GLIS2 | GLIS family zinc finger 2(GLIS2) | -0.5328 | 0.0001 | 0.0047 |
| GLMP | glycosylated lysosomal membrane protein (GLMP) | -0.7173 | 0.0000 | 0.0000 |
| GLS | glutaminase (GLS) | 0.6049 | 0.0002 | 0.0073 |
| GM2A | GM2 ganglioside activator (GM2A) | -0.6160 | 0.0003 | 0.0111 |
| GNAI2 | G protein subunit alpha i2(GNAI2) | -0.4145 | 0.0004 | 0.0142 |
| GNB4 | G protein subunit beta 4(GNB4) | 0.3714 | 0.0020 | 0.0442 |
| GOLGA8M | golgin A8 family member M(GOLGA8M) | 2.9296 | 0.0009 | 0.0242 |
| GPAM | glycerol-3-phosphate acyltransferase, mitochondrial (GPAM) | 1.1280 | 0.0000 | 0.0001 |
| GPAT2 | glycerol-3-phosphate acyltransferase 2, mitochondrial (GPAT2) | 4.3310 | 0.0000 | 0.0000 |
| GPC1 | glypican 1(GPC1) | -1.1438 | 0.0000 | 0.0000 |
| GPC4 | glypican 4(GPC4) | 1.9593 | 0.0005 | 0.0165 |
| GPCPD1 | glycerophosphocholine phosphodiesterase 1(GPCPD1) | 0.5561 | 0.0000 | 0.0000 |
| GPM6B | glycoprotein M6B(GPM6B) | 4.2371 | 0.0022 | 0.0467 |
| GPR176 | G protein-coupled receptor 176(GPR176) | -0.5145 | 0.0003 | 0.0122 |
| GPR183 | G protein-coupled receptor 183(GPR183) | -2.2565 | 0.0006 | 0.0189 |
| GPT2 | glutamic--pyruvic transaminase 2(GPT2) | 0.6465 | 0.0016 | 0.0384 |
| GPX3 | glutathione peroxidase 3(GPX3) | 1.3889 | 0.0006 | 0.0186 |
| GREM2 | gremlin 2, DAN family BMP antagonist (GREM2) | -0.9529 | 0.0000 | 0.0000 |
| GSC | goosecoid homeobox (GSC) | 2.7325 | 0.0005 | 0.0171 |
| GTF2E1 | general transcription factor IIE subunit 1(GTF2E1) | 0.8542 | 0.0007 | 0.0198 |
| H2AC19 | H2A Clustered Histone 19 | -1.0675 | 0.0000 | 0.0002 |
| H3-2 | H3.2 Histone (Putative) | 1.1051 | 0.0001 | 0.0058 |
| HACD4 | 3-hydroxyacyl-CoA dehydratase 4(HACD4) | -0.7573 | 0.0000 | 0.0004 |
| HDAC10 | histone deacetylase 10(HDAC10) | -0.5418 | 0.0003 | 0.0102 |
| HDAC5 | histone deacetylase 5(HDAC5) | -0.5368 | 0.0000 | 0.0005 |
| HIF1A | hypoxia inducible factor 1 alpha subunit (HIF1A) | 0.7686 | 0.0003 | 0.0104 |
| HIVEP1 | human immunodeficiency virus type I enhancer binding protein 1(HIVEP1) | 0.5699 | 0.0009 | 0.0248 |
| HLA-B | major histocompatibility complex, class I, B(HLA-B) | -1.1355 | 0.0000 | 0.0001 |
| HLA-C | major histocompatibility complex, class I, C(HLA-C) | -0.9217 | 0.0017 | 0.0390 |
| HLA-E | major histocompatibility complex, class I, E(HLA-E) | -0.6173 | 0.0001 | 0.0028 |
| HMGA1 | high mobility group AT-hook 1(HMGA1) | -0.8551 | 0.0022 | 0.0471 |
| HNMT | histamine N-methyltransferase (HNMT) | 0.9497 | 0.0000 | 0.0000 |
| HNRNPA0 | heterogeneous nuclear ribonucleoprotein A0(HNRNPA0) | 0.4307 | 0.0000 | 0.0004 |
| HNRNPF | heterogeneous nuclear ribonucleoprotein F(HNRNPF) | 0.3871 | 0.0015 | 0.0358 |
| HOXD3 | homeobox D3(HOXD3) | 1.0292 | 0.0000 | 0.0012 |
| HSBP1 | heat shock factor binding protein 1(HSBP1) | -0.4605 | 0.0000 | 0.0009 |
| HSD3B7 | hydroxy-delta-5-steroid dehydrogenase, 3 beta- and steroid delta-isomerase 7(HSD3B7) | -0.9530 | 0.0007 | 0.0212 |
| HSP90AA1 | heat shock protein 90 alpha family class A member 1(HSP90AA1) | -0.2839 | 0.0019 | 0.0429 |
| HSPA9 | heat shock protein family A (Hsp70) member 9(HSPA9) | 0.4581 | 0.0000 | 0.0000 |
| HSPB2 | heat shock protein family B (small) member 2(HSPB2) | -0.4951 | 0.0003 | 0.0100 |
| HSPB3 | heat shock protein family B (small) member 3(HSPB3) | -2.5316 | 0.0000 | 0.0001 |
| HTR2A | 5-hydroxytryptamine receptor 2A(HTR2A) | 1.2348 | 0.0020 | 0.0450 |
| HUNK | hormonally up-regulated Neu-associated kinase (HUNK) | 1.5743 | 0.0021 | 0.0464 |
| IARS1 | Isoleucyl-TRNA Synthetase 1 | 0.5285 | 0.0000 | 0.0027 |
| ICAM5 | intercellular adhesion molecule 5(ICAM5) | -1.6126 | 0.0000 | 0.0026 |
| ICMT | isoprenylcysteine carboxyl methyltransferase (ICMT) | -0.4293 | 0.0006 | 0.0189 |
| IDS | iduronate 2-sulfatase (IDS) | -0.6541 | 0.0000 | 0.0023 |
| IFFO1 | intermediate filament family orphan 1(IFFO1) | -1.0338 | 0.0000 | 0.0002 |
| IFI44L | interferon induced protein 44 like (IFI44L) | 1.4548 | 0.0023 | 0.0490 |
| IGF1 | insulin like growth factor 1(IGF1) | 5.0628 | 0.0000 | 0.0005 |
| IGFBP6 | insulin like growth factor binding protein 6(IGFBP6) | -1.1693 | 0.0001 | 0.0047 |
| IGIP | IgA inducing protein (IGIP) | -0.6425 | 0.0004 | 0.0141 |
| IL17RB | interleukin 17 receptor B(IL17RB) | -2.5456 | 0.0001 | 0.0055 |
| IL20RB | interleukin 20 receptor subunit beta (IL20RB) | -0.7074 | 0.0002 | 0.0092 |
| IL21R | interleukin 21 receptor (IL21R) | 1.4041 | 0.0000 | 0.0002 |
| IMPACT | impact RWD domain protein (IMPACT) | 0.4420 | 0.0001 | 0.0038 |
| INAFM2 | InaF motif containing 2(INAFM2) | -0.7496 | 0.0001 | 0.0056 |
| INHBE | inhibin beta E subunit (INHBE) | 2.4445 | 0.0000 | 0.0000 |
| IRS2 | insulin receptor substrate 2(IRS2) | 0.8275 | 0.0000 | 0.0009 |
| ITGA3 | integrin subunit alpha 3(ITGA3) | -1.2113 | 0.0000 | 0.0004 |
| ITPKC | inositol-trisphosphate 3-kinase C(ITPKC) | -0.7535 | 0.0001 | 0.0036 |
| ITPR3 | inositol 1,4,5-trisphosphate receptor type 3(ITPR3) | -0.6587 | 0.0003 | 0.0103 |
| JADE1 | jade family PHD finger 1(JADE1) | 0.6865 | 0.0001 | 0.0049 |
| JMJD8 | jumonji domain containing 8(JMJD8) | -0.4651 | 0.0021 | 0.0466 |
| JUN | Jun proto-oncogene, AP-1 transcription factor subunit (JUN) | 0.8012 | 0.0021 | 0.0461 |
| KANK2 | KN motif and ankyrin repeat domains 2(KANK2) | -0.3297 | 0.0009 | 0.0244 |
| KCNA4 | potassium voltage-gated channel subfamily A member 4(KCNA4) | 4.5286 | 0.0010 | 0.0262 |
| KCNB1 | potassium voltage-gated channel subfamily B member 1(KCNB1) | -3.3101 | 0.0000 | 0.0002 |
| KCNE3 | potassium voltage-gated channel subfamily E regulatory subunit 3(KCNE3) | 1.7444 | 0.0005 | 0.0166 |
| KCNG1 | potassium voltage-gated channel modifier subfamily G member 1(KCNG1) | 1.1120 | 0.0001 | 0.0062 |
| KCNIP3 | potassium voltage-gated channel interacting protein 3(KCNIP3) | -0.9522 | 0.0002 | 0.0067 |
| KCNJ8 | potassium voltage-gated channel subfamily J member 8(KCNJ8) | 2.6664 | 0.0000 | 0.0016 |
| KCNS1 | potassium voltage-gated channel modifier subfamily S member 1(KCNS1) | -2.1554 | 0.0001 | 0.0034 |
| KIAA1324L | KIAA1324 like (KIAA1324L) | 2.6763 | 0.0000 | 0.0008 |
| KIAA1549 | KIAA1549(KIAA1549) | -1.2872 | 0.0000 | 0.0000 |
| KIAA1549L | KIAA1549 like (KIAA1549L) | -0.9136 | 0.0002 | 0.0077 |
| KIF13A | kinesin family member 13A(KIF13A) | -0.5468 | 0.0000 | 0.0000 |
| KIF1C | kinesin family member 1C(KIF1C) | -0.7860 | 0.0000 | 0.0000 |
| KIF3B | kinesin family member 3B(KIF3B) | -0.4785 | 0.0011 | 0.0283 |
| KIRREL3 | kin of IRRE like 3 (Drosophila)(KIRREL3) | -1.6119 | 0.0000 | 0.0000 |
| KLF14 | Kruppel like factor 14(KLF14) | 4.5977 | 0.0002 | 0.0082 |
| KLF7 | Kruppel like factor 7(KLF7) | 0.5454 | 0.0003 | 0.0111 |
| KLHDC7B | kelch domain containing 7B(KLHDC7B) | 1.9449 | 0.0009 | 0.0252 |
| KLHL8 | kelch like family member 8(KLHL8) | 0.6014 | 0.0007 | 0.0215 |
| KRT15 | keratin 15(KRT15) | -2.6166 | 0.0013 | 0.0315 |
| KRT7 | keratin 7(KRT7) | 3.9203 | 0.0001 | 0.0042 |
| KRTAP1-5 | keratin associated protein 1-5(KRTAP1-5) | -2.2211 | 0.0000 | 0.0004 |
| LAMA5 | laminin subunit alpha 5(LAMA5) | -1.1729 | 0.0005 | 0.0161 |
| LAMTOR4 | late endosomal/lysosomal adaptor, MAPK and MTOR activator 4(LAMTOR4) | -0.3282 | 0.0021 | 0.0460 |
| LARS1 | Leucyl-TRNA Synthetase 1 | 0.3803 | 0.0005 | 0.0161 |
| LCTL | lactase like (LCTL) | 2.4577 | 0.0001 | 0.0062 |
| LDHB | lactate dehydrogenase B(LDHB) | 0.4703 | 0.0001 | 0.0034 |
| LDLRAP1 | low density lipoprotein receptor adaptor protein 1(LDLRAP1) | -0.4097 | 0.0005 | 0.0164 |
| LGR4 | leucine rich repeat containing G protein-coupled receptor 4(LGR4) | 1.2106 | 0.0014 | 0.0350 |
| LIMD2 | LIM domain containing 2(LIMD2) | 0.7651 | 0.0001 | 0.0048 |
| LINC00578 | long intergenic non-protein coding RNA 578(LINC00578) | 3.3227 | 0.0001 | 0.0029 |
| LINC01085 | long intergenic non-protein coding RNA 1085(LINC01085) | -4.9460 | 0.0000 | 0.0000 |
| LINC01145 | Long Intergenic Non-Protein Coding RNA 1145 | 1.3515 | 0.0000 | 0.0001 |
| LINC01503 | long intergenic non-protein coding RNA 1503(LINC01503) | 1.4395 | 0.0001 | 0.0062 |
| LINC01515 | long intergenic non-protein coding RNA 1515(LINC01515) | -3.6335 | 0.0001 | 0.0057 |
| LINC01638 | Long Intergenic Non-Protein Coding RNA 1638 | 1.5917 | 0.0003 | 0.0102 |
| LINC01936 | Long Intergenic Non-Protein Coding RNA 1936 | 1.4080 | 0.0018 | 0.0420 |
| LIPG | lipase G, endothelial type (LIPG) | 2.4285 | 0.0017 | 0.0389 |
| LMF1 | lipase maturation factor 1(LMF1) | -0.5826 | 0.0004 | 0.0126 |
| LMNA | lamin A/C(LMNA) | -0.4626 | 0.0012 | 0.0306 |
| LMO4 | LIM domain only 4(LMO4) | 0.8049 | 0.0006 | 0.0188 |
| LONP1 | lon peptidase 1, mitochondrial (LONP1) | 0.5523 | 0.0000 | 0.0027 |
| LOXL1 | lysyl oxidase like 1(LOXL1) | -0.6649 | 0.0000 | 0.0000 |
| LOXL4 | lysyl oxidase like 4(LOXL4) | -1.8183 | 0.0000 | 0.0011 |
| LRMDA | Leucine Rich Melanocyte Differentiation Associated | 1.1596 | 0.0010 | 0.0269 |
| LRPAP1 | LDL receptor related protein associated protein 1(LRPAP1) | -0.7858 | 0.0009 | 0.0260 |
| LRRFIP2 | LRR binding FLII interacting protein 2(LRRFIP2) | -0.7314 | 0.0009 | 0.0259 |
| LRRN4CL | LRRN4 C-terminal like (LRRN4CL) | -1.2111 | 0.0000 | 0.0000 |
| LY6E | lymphocyte antigen 6 complex, locus E(LY6E) | -1.0571 | 0.0005 | 0.0166 |
| LYNX1 | Ly6/neurotoxin 1(LYNX1) | -1.1334 | 0.0015 | 0.0365 |
| LYPD3 | LY6/PLAUR domain containing 3(LYPD3) | -2.6550 | 0.0000 | 0.0016 |
| LYPD6 | LY6/PLAUR domain containing 6(LYPD6) | -1.5093 | 0.0001 | 0.0043 |
| LYPD6B | LY6/PLAUR domain containing 6B(LYPD6B) | -1.8648 | 0.0000 | 0.0001 |
| LYPLA1 | lysophospholipase I(LYPLA1) | 0.5221 | 0.0022 | 0.0470 |
| MALAT1 | metastasis associated lung adenocarcinoma transcript 1 (non-protein coding) (MALAT1) | -0.9479 | 0.0000 | 0.0000 |
| MAP2K3 | mitogen-activated protein kinase kinase 3(MAP2K3) | -0.5995 | 0.0000 | 0.0005 |
| MAPKAPK3 | mitogen-activated protein kinase-activated protein kinase 3(MAPKAPK3) | -0.4868 | 0.0003 | 0.0095 |
| MARK1 | microtubule affinity regulating kinase 1(MARK1) | 3.5625 | 0.0009 | 0.0258 |
| MARS1 | Methionyl-TRNA Synthetase 1 | 0.5383 | 0.0000 | 0.0026 |
| MATN2 | matrilin 2(MATN2) | -0.9794 | 0.0000 | 0.0006 |
| MBD4 | methyl-CpG binding domain 4, DNA glycosylase (MBD4) | 0.5041 | 0.0003 | 0.0111 |
| MBNL1-AS1 | MBNL1 antisense RNA 1(MBNL1-AS1) | -0.5080 | 0.0016 | 0.0378 |
| MBOAT7 | membrane bound O-acyltransferase domain containing 7(MBOAT7) | -0.6432 | 0.0003 | 0.0102 |
| MDFI | MyoD family inhibitor (MDFI) | 6.6073 | 0.0000 | 0.0001 |
| MEF2A | myocyte enhancer factor 2A(MEF2A) | -0.5554 | 0.0001 | 0.0052 |
| MFAP3L | microfibrillar associated protein 3 like (MFAP3L) | 1.2370 | 0.0020 | 0.0439 |
| MFAP4 | microfibrillar associated protein 4(MFAP4) | 0.9426 | 0.0002 | 0.0075 |
| MFSD10 | major facilitator superfamily domain containing 10(MFSD10) | -0.5952 | 0.0002 | 0.0091 |
| MFSD12 | major facilitator superfamily domain containing 12(MFSD12) | -0.5394 | 0.0014 | 0.0353 |
| MFSD6 | major facilitator superfamily domain containing 6(MFSD6) | -1.1723 | 0.0008 | 0.0233 |
| MGARP | mitochondria localized glutamic acid rich protein (MGARP) | -0.7715 | 0.0005 | 0.0172 |
| MGAT4B | mannosyl (alpha-1,3-)-glycoprotein beta-1,4-N-acetylglucosaminyltransferase, isozyme B(MGAT4B) | -0.5231 | 0.0001 | 0.0047 |
| MIER3 | MIER family member 3(MIER3) | 0.5910 | 0.0007 | 0.0210 |
| MIR503HG | MIR503 host gene (MIR503HG) | 1.9294 | 0.0001 | 0.0034 |
| MIR99AHG | mir-99a-let-7c cluster host gene (MIR99AHG) | 1.3152 | 0.0000 | 0.0002 |
| MME | membrane metalloendopeptidase (MME) | 1.2066 | 0.0001 | 0.0029 |
| MMP24OS | MMP24 Opposite Strand | -0.5785 | 0.0000 | 0.0001 |
| MOK | MOK protein kinase (MOK) | -1.8993 | 0.0000 | 0.0000 |
| MT-ATP6 | Mitochondrially Encoded ATP Synthase Membrane Subunit 6 | -0.6422 | 0.0002 | 0.0093 |
| MTATP6P1 | mitochondrially encoded ATP synthase 6 pseudogene 1(MTATP6P1) | -0.7252 | 0.0007 | 0.0207 |
| MT-CO1 | Mitochondrially Encoded Cytochrome C Oxidase I | -0.5153 | 0.0000 | 0.0022 |
| MTCO1P12 | mitochondrially encoded cytochrome c oxidase I pseudogene 12(MTCO1P12) | -0.5331 | 0.0014 | 0.0352 |
| MT-CO2 | Mitochondrially Encoded Cytochrome C Oxidase II | -0.8283 | 0.0002 | 0.0086 |
| MTHFD2 | methylenetetrahydrofolate dehydrogenase (NADP+ dependent) 2, methenyltetrahydrofolate cyclohydrolase (MTHFD2) | 1.0596 | 0.0000 | 0.0000 |
| MT-ND1 | Mitochondrially Encoded NADH: Ubiquinone Oxidoreductase Core Subunit 1 | -0.5235 | 0.0000 | 0.0023 |
| MT-ND2 | Mitochondrially Encoded NADH: Ubiquinone Oxidoreductase Core Subunit 2 | -0.6281 | 0.0017 | 0.0393 |
| MTND2P28 | mitochondrially encoded NADH: ubiquinone oxidoreductase core subunit 2 pseudogene 28(MTND2P28) | -0.6876 | 0.0010 | 0.0264 |
| MT-ND3 | Mitochondrially Encoded NADH: Ubiquinone Oxidoreductase Core Subunit 3 | -0.6251 | 0.0008 | 0.0219 |
| MT-ND5 | Mitochondrially Encoded NADH: Ubiquinone Oxidoreductase Core Subunit 5 | -0.6271 | 0.0000 | 0.0015 |
| MT-RNR1 | Mitochondrially Encoded 12S RRNA | -1.1473 | 0.0000 | 0.0001 |
| MT-RNR2 | Mitochondrially Encoded 16S RRNA | -0.4799 | 0.0009 | 0.0259 |
| MTSS1 | MTSS1, I-BAR domain containing (MTSS1) | -1.2511 | 0.0008 | 0.0228 |
| MUC20-OT1 | MUC20 Overlapping Transcript | -0.6715 | 0.0002 | 0.0093 |
| MXRA8 | matrix remodeling associated 8(MXRA8) | -0.4727 | 0.0003 | 0.0102 |
| MYBL1 | MYB proto-oncogene like 1(MYBL1) | -1.5627 | 0.0000 | 0.0002 |
| MYNN | myoneurin (MYNN) | 0.4356 | 0.0020 | 0.0445 |
| MYO18A | myosin XVIIIA(MYO18A) | -0.4814 | 0.0016 | 0.0375 |
| MYPN | myopalladin (MYPN) | -2.7570 | 0.0001 | 0.0056 |
| NAALADL2 | N-acetylated alpha-linked acidic dipeptidase like 2(NAALADL2) | -0.9212 | 0.0000 | 0.0012 |
| NAGA | alpha-N-acetylgalactosaminidase (NAGA) | -0.4198 | 0.0018 | 0.0414 |
| NARS1 | Asparaginyl-TRNA Synthetase 1 | 0.3667 | 0.0016 | 0.0384 |
| NBL1 | neuroblastoma 1, DAN family BMP antagonist (NBL1) | -1.2085 | 0.0000 | 0.0000 |
| NCEH1 | neutral cholesterol ester hydrolase 1(NCEH1) | -1.3901 | 0.0000 | 0.0002 |
| NCOA7 | nuclear receptor coactivator 7(NCOA7) | 0.5506 | 0.0023 | 0.0495 |
| NCS1 | neuronal calcium sensor 1(NCS1) | -0.5199 | 0.0000 | 0.0001 |
| NEDD4 | neural precursor cell expressed, developmentally down-regulated 4, E3 ubiquitin protein ligase (NEDD4) | 0.4057 | 0.0023 | 0.0488 |
| NEFL | neurofilament, light polypeptide (NEFL) | 3.2710 | 0.0004 | 0.0126 |
| NENF | neudesin neurotrophic factor (NENF) | -0.4056 | 0.0002 | 0.0067 |
| NF2 | neurofibromin 2(NF2) | -0.4780 | 0.0002 | 0.0073 |
| NFASC | neurofascin (NFASC) | -0.6991 | 0.0000 | 0.0016 |
| NFATC2 | nuclear factor of activated T-cells 2(NFATC2) | -3.4974 | 0.0000 | 0.0000 |
| NFKB2 | nuclear factor kappa B subunit 2(NFKB2) | -0.4036 | 0.0020 | 0.0456 |
| NGEF | neuronal guanine nucleotide exchange factor (NGEF) | -5.6823 | 0.0000 | 0.0001 |
| NIN | ninein (NIN) | -0.4601 | 0.0008 | 0.0224 |
| NIPAL2 | NIPA like domain containing 2(NIPAL2) | -0.7043 | 0.0000 | 0.0010 |
| NIPAL3 | NIPA like domain containing 3(NIPAL3) | -1.2133 | 0.0000 | 0.0000 |
| NLRP10 | NLR family pyrin domain containing 10(NLRP10) | -2.2935 | 0.0000 | 0.0001 |
| NOLC1 | nucleolar and coiled-body phosphoprotein 1(NOLC1) | 0.5939 | 0.0000 | 0.0016 |
| NOS3 | nitric oxide synthase 3(NOS3) | 2.5281 | 0.0012 | 0.0303 |
| NOTCH3 | notch 3(NOTCH3) | -1.3878 | 0.0007 | 0.0205 |
| NOX4 | NADPH oxidase 4(NOX4) | 1.5817 | 0.0003 | 0.0102 |
| NPDC1 | neural proliferation, differentiation, and control 1(NPDC1) | -0.8566 | 0.0010 | 0.0263 |
| NPTX1 | neuronal pentraxin 1(NPTX1) | -1.7373 | 0.0000 | 0.0003 |
| NR2F1 | nuclear receptor subfamily 2 group F member 1(NR2F1) | 1.0049 | 0.0010 | 0.0271 |
| NRN1 | neuritin 1(NRN1) | -0.6493 | 0.0000 | 0.0002 |
| NRP2 | neuropilin 2(NRP2) | 1.0733 | 0.0002 | 0.0078 |
| NT5E | 5'-nucleotidase ecto (NT5E) | -1.1556 | 0.0000 | 0.0003 |
| NTRK3 | neurotrophic receptor tyrosine kinase 3(NTRK3) | -3.3315 | 0.0001 | 0.0043 |
| NUDT21 | nudix hydrolase 21(NUDT21) | 0.3692 | 0.0006 | 0.0185 |
| NUMBL | NUMB like, endocytic adaptor protein (NUMBL) | -0.4174 | 0.0005 | 0.0173 |
| NUP98 | nucleoporin 98(NUP98) | 0.3398 | 0.0016 | 0.0375 |
| OIP5-AS1 | OIP5 antisense RNA 1(OIP5-AS1) | 0.5225 | 0.0000 | 0.0006 |
| ORAI3 | ORAI calcium release-activated calcium modulator 3(ORAI3) | -0.7194 | 0.0005 | 0.0156 |
| OSBP2 | oxysterol binding protein 2(OSBP2) | -2.3399 | 0.0000 | 0.0004 |
| PAM | peptidylglycine alpha-amidating monooxygenase (PAM) | -0.9676 | 0.0005 | 0.0167 |
| PARD3B | par-3 family cell polarity regulator beta (PARD3B) | -0.7846 | 0.0000 | 0.0001 |
| PARP8 | poly (ADP-ribose) polymerase family member 8(PARP8) | 3.1539 | 0.0021 | 0.0464 |
| PBX1 | PBX homeobox 1(PBX1) | 0.7722 | 0.0000 | 0.0023 |
| PCDH9 | protocadherin 9(PCDH9) | 1.7627 | 0.0000 | 0.0000 |
| PCDHGB6 | protocadherin gamma subfamily B, 6(PCDHGB6) | 1.4370 | 0.0002 | 0.0082 |
| PCK2 | phosphoenolpyruvate carboxykinase 2, mitochondrial (PCK2) | 0.9622 | 0.0000 | 0.0000 |
| PCSK1 | proprotein convertase subtilisin/kexin type 1(PCSK1) | 2.4307 | 0.0001 | 0.0064 |
| PCSK5 | proprotein convertase subtilisin/kexin type 5(PCSK5) | 2.1519 | 0.0000 | 0.0000 |
| PCYOX1 | prenylcysteine oxidase 1(PCYOX1) | -0.7995 | 0.0002 | 0.0076 |
| PDGFB | platelet derived growth factor subunit B(PDGFB) | 5.2079 | 0.0007 | 0.0198 |
| PDGFD | platelet derived growth factor D(PDGFD) | 1.4314 | 0.0000 | 0.0000 |
| PEX6 | peroxisomal biogenesis factor 6(PEX6) | 0.5051 | 0.0010 | 0.0262 |
| PGAP6 | Post-Glycosylphosphatidylinositol Attachment To Proteins 6 | -0.4879 | 0.0002 | 0.0089 |
| PHACTR3 | phosphatase and actin regulator 3(PHACTR3) | 4.1572 | 0.0003 | 0.0100 |
| PHGDH | phosphoglycerate dehydrogenase (PHGDH) | 0.6867 | 0.0001 | 0.0043 |
| PI16 | peptidase inhibitor 16(PI16) | 3.4013 | 0.0018 | 0.0408 |
| PI4KAP2 | phosphatidylinositol 4-kinase alpha pseudogene 2(PI4KAP2) | -0.9526 | 0.0000 | 0.0004 |
| PICSAR | P38 inhibited cutaneous squamous cell carcinoma associated lincRNA (PICSAR) | 5.9841 | 0.0009 | 0.0259 |
| PIK3R3 | phosphoinositide-3-kinase regulatory subunit 3(PIK3R3) | -0.9867 | 0.0001 | 0.0064 |
| PJA1 | praja ring finger ubiquitin ligase 1(PJA1) | 0.5068 | 0.0011 | 0.0279 |
| PKD2 | polycystin 2, transient receptor potential cation channel (PKD2) | -0.7511 | 0.0004 | 0.0141 |
| PKIG | protein kinase (cAMP-dependent, catalytic) inhibitor gamma (PKIG) | -0.9822 | 0.0010 | 0.0263 |
| PKN1 | protein kinase N1(PKN1) | -0.3744 | 0.0012 | 0.0296 |
| PLA2G15 | phospholipase A2 group XV(PLA2G15) | -0.9474 | 0.0000 | 0.0000 |
| PLA2R1 | phospholipase A2 receptor 1(PLA2R1) | -0.6453 | 0.0015 | 0.0369 |
| PLAC1 | placenta specific 1(PLAC1) | 4.3143 | 0.0012 | 0.0315 |
| PLCD1 | phospholipase C delta 1(PLCD1) | -0.7652 | 0.0000 | 0.0004 |
| PLEC | plectin (PLEC) | -0.7812 | 0.0000 | 0.0011 |
| PLP2 | proteolipid protein 2(PLP2) | -0.6442 | 0.0016 | 0.0381 |
| PLPP2 | phospholipid phosphatase 2(PLPP2) | 4.0119 | 0.0001 | 0.0046 |
| PLPP4 | phospholipid phosphatase 4(PLPP4) | 1.0814 | 0.0000 | 0.0000 |
| PLS3 | plastin 3(PLS3) | 0.5549 | 0.0001 | 0.0042 |
| PLSCR1 | phospholipid scramblase 1(PLSCR1) | 0.7500 | 0.0000 | 0.0018 |
| PLXDC2 | plexin domain containing 2(PLXDC2) | 4.2060 | 0.0007 | 0.0205 |
| PNPLA6 | patatin like phospholipase domain containing 6(PNPLA6) | -0.6430 | 0.0001 | 0.0028 |
| PODN | podocan (PODN) | -0.9085 | 0.0001 | 0.0061 |
| POLR2L | RNA polymerase II subunit L(POLR2L) | -0.6071 | 0.0000 | 0.0001 |
| POMGNT1 | protein O-linked mannose N-acetylglucosaminyltransferase 1 (beta 1,2-) (POMGNT1) | -0.6554 | 0.0008 | 0.0224 |
| PPFIBP2 | PPFIA binding protein 2(PPFIBP2) | 0.7037 | 0.0000 | 0.0014 |
| PPP1R12A | protein phosphatase 1 regulatory subunit 12A(PPP1R12A) | 0.3686 | 0.0018 | 0.0417 |
| PPP3CA | protein phosphatase 3 catalytic subunit alpha (PPP3CA) | 0.4205 | 0.0004 | 0.0134 |
| PRELP | proline and arginine rich end leucine rich repeat protein (PRELP) | -1.9811 | 0.0000 | 0.0020 |
| PREX1 | phosphatidylinositol-3,4,5-trisphosphate dependent Rac exchange factor 1(PREX1) | 1.0772 | 0.0017 | 0.0390 |
| PRICKLE1 | prickle planar cell polarity protein 1(PRICKLE1) | 2.2404 | 0.0017 | 0.0397 |
| PRICKLE2 | prickle planar cell polarity protein 2(PRICKLE2) | 0.8628 | 0.0013 | 0.0323 |
| PRIM2 | primase (DNA) subunit 2(PRIM2) | -0.6940 | 0.0004 | 0.0135 |
| PRKAB2 | protein kinase AMP-activated non-catalytic subunit beta 2(PRKAB2) | 0.5542 | 0.0000 | 0.0002 |
| PRL | prolactin (PRL) | 4.3352 | 0.0011 | 0.0279 |
| PRNP | prion protein (PRNP) | -0.6324 | 0.0021 | 0.0467 |
| PRPH2 | peripherin 2(PRPH2) | -1.0871 | 0.0000 | 0.0026 |
| PRPS1 | phosphoribosyl pyrophosphate synthetase 1(PRPS1) | 1.0850 | 0.0000 | 0.0014 |
| PRR7 | proline rich 7, synaptic (PRR7) | -0.7646 | 0.0010 | 0.0262 |
| PRSS12 | protease, serine 12(PRSS12) | -1.2755 | 0.0002 | 0.0082 |
| PRXL2A | Peroxiredoxin Like 2A | 4.6276 | 0.0008 | 0.0223 |
| PSAT1 | phosphoserine aminotransferase 1(PSAT1) | 0.9058 | 0.0000 | 0.0000 |
| PSEN2 | presenilin 2(PSEN2) | -1.0165 | 0.0023 | 0.0488 |
| PSG2 | pregnancy specific beta-1-glycoprotein 2(PSG2) | -2.6682 | 0.0000 | 0.0000 |
| PSG9 | pregnancy specific beta-1-glycoprotein 9(PSG9) | -2.3971 | 0.0007 | 0.0198 |
| PSMB2 | proteasome subunit beta 2(PSMB2) | 0.3780 | 0.0007 | 0.0198 |
| PSPH | phosphoserine phosphatase (PSPH) | 0.6097 | 0.0008 | 0.0228 |
| PTGES | prostaglandin E synthase (PTGES) | -0.8495 | 0.0011 | 0.0279 |
| PTPRU | protein tyrosine phosphatase, receptor type U(PTPRU) | -0.7977 | 0.0002 | 0.0092 |
| PUM3 | pumilio RNA binding family member 3(PUM3) | 0.3802 | 0.0019 | 0.0438 |
| RAB11FIP3 | RAB11 family interacting protein 3(RAB11FIP3) | -0.5959 | 0.0000 | 0.0006 |
| RAB15 | RAB15, member RAS oncogene family (RAB15) | 0.7355 | 0.0001 | 0.0054 |
| RAB27B | RAB27B, member RAS oncogene family (RAB27B) | -2.0112 | 0.0001 | 0.0056 |
| RABAC1 | Rab acceptor 1(RABAC1) | -0.3043 | 0.0014 | 0.0348 |
| RABGEF1 | RAB guanine nucleotide exchange factor 1(RABGEF1) | -0.4452 | 0.0001 | 0.0051 |
| RAD21 | RAD21 cohesin complex component (RAD21) | 0.3608 | 0.0003 | 0.0112 |
| RAF1 | Raf-1 proto-oncogene, serine/threonine kinase (RAF1) | -0.3560 | 0.0011 | 0.0278 |
| RAI14 | retinoic acid induced 14(RAI14) | 0.4261 | 0.0007 | 0.0211 |
| RAMP1 | receptor activity modifying protein 1(RAMP1) | -2.1215 | 0.0000 | 0.0009 |
| RANBP17 | RAN binding protein 17(RANBP17) | 3.3369 | 0.0022 | 0.0484 |
| RAPGEF1 | Rap guanine nucleotide exchange factor 1(RAPGEF1) | -0.5350 | 0.0003 | 0.0111 |
| RASA2 | RAS p21 protein activator 2(RASA2) | -0.6412 | 0.0000 | 0.0004 |
| RASSF3 | Ras association domain family member 3(RASSF3) | -0.4318 | 0.0008 | 0.0229 |
| RASSF8-AS1 | RASSF8 antisense RNA 1(RASSF8-AS1) | -0.5459 | 0.0007 | 0.0211 |
| RBPMS2 | RNA binding protein with multiple splicing 2(RBPMS2) | 2.8402 | 0.0000 | 0.0004 |
| RCOR2 | REST corepressor 2(RCOR2) | 1.5186 | 0.0006 | 0.0180 |
| RDH5 | retinol dehydrogenase 5(RDH5) | -2.2552 | 0.0000 | 0.0000 |
| REC8 | REC8 meiotic recombination protein (REC8) | -0.8628 | 0.0001 | 0.0061 |
| REEP2 | receptor accessory protein 2(REEP2) | -0.7837 | 0.0001 | 0.0048 |
| REEP5 | receptor accessory protein 5(REEP5) | -0.4489 | 0.0001 | 0.0043 |
| REPS2 | RALBP1 associated Eps domain containing 2(REPS2) | 2.8756 | 0.0004 | 0.0126 |
| RETSAT | retinol saturase (RETSAT) | -0.7231 | 0.0000 | 0.0002 |
| RFX8 | RFX family member 8, lacking RFX DNA binding domain (RFX8) | -1.4530 | 0.0001 | 0.0058 |
| RGCC | regulator of cell cycle (RGCC) | -1.5791 | 0.0007 | 0.0210 |
| RGMB | repulsive guidance molecule family member b(RGMB) | -1.3325 | 0.0000 | 0.0000 |
| RGS20 | regulator of G-protein signaling 20(RGS20) | -1.5956 | 0.0002 | 0.0082 |
| RHOQ | ras homolog family member Q(RHOQ) | -0.3969 | 0.0021 | 0.0467 |
| RIMKLB | ribosomal modification protein rimK like family member B(RIMKLB) | 0.4632 | 0.0009 | 0.0242 |
| RIN1 | Ras and Rab interactor 1(RIN1) | -0.7982 | 0.0001 | 0.0061 |
| RIPOR2 | RHO Family Interacting Cell Polarization Regulator 2 | -1.7306 | 0.0000 | 0.0000 |
| RMI1 | RecQ mediated genome instability 1(RMI1) | 0.7358 | 0.0019 | 0.0425 |
| RMND5A | required for meiotic nuclear division 5 homolog A(RMND5A) | 0.6002 | 0.0001 | 0.0031 |
| RNASE4 | ribonuclease A family member 4(RNASE4) | -0.8012 | 0.0009 | 0.0244 |
| RNASEK | ribonuclease K(RNASEK) | -0.3427 | 0.0013 | 0.0323 |
| RNF150 | ring finger protein 150(RNF150) | -0.6598 | 0.0001 | 0.0041 |
| RNY3 | RNA, Ro-associated Y3(RNY3) | -22.8998 | 0.0000 | 0.0000 |
| RPL13P12 | ribosomal protein L13 pseudogene 12(RPL13P12) | -2.6700 | 0.0000 | 0.0018 |
| RPS10-NUDT3 | RPS10-NUDT3 readthrough (RPS10-NUDT3) | 0.5874 | 0.0005 | 0.0168 |
| RRAD | RRAD, Ras related glycolysis inhibitor and calcium channel regulator (RRAD) | -1.1819 | 0.0005 | 0.0166 |
| RRAS | related RAS viral (r-ras) oncogene homolog (RRAS) | -0.6678 | 0.0000 | 0.0000 |
| RTN4 | reticulon 4(RTN4) | -0.6114 | 0.0000 | 0.0018 |
| S100A10 | S100 calcium binding protein A10(S100A10) | -0.9511 | 0.0000 | 0.0000 |
| S100A13 | S100 calcium binding protein A13(S100A13) | -0.4506 | 0.0000 | 0.0003 |
| S100A6 | S100 calcium binding protein A6(S100A6) | -0.6903 | 0.0008 | 0.0240 |
| S1PR3 | sphingosine-1-phosphate receptor 3(S1PR3) | -1.1626 | 0.0000 | 0.0001 |
| SAMD11 | sterile alpha motif domain containing 11(SAMD11) | 1.3825 | 0.0000 | 0.0005 |
| SARS1 | Seryl-TRNA Synthetase 1 | 0.3757 | 0.0000 | 0.0015 |
| SAV1 | salvador family WW domain containing protein 1(SAV1) | -0.6245 | 0.0001 | 0.0048 |
| SCAMP5 | secretory carrier membrane protein 5(SCAMP5) | 1.6373 | 0.0000 | 0.0000 |
| SCARB1 | scavenger receptor class B member 1(SCARB1) | -0.6526 | 0.0001 | 0.0061 |
| SCCPDH | saccharopine dehydrogenase (putative)(SCCPDH) | -0.3695 | 0.0001 | 0.0048 |
| SDF4 | stromal cell derived factor 4(SDF4) | -0.6114 | 0.0002 | 0.0078 |
| SEC23IP | SEC23 interacting protein (SEC23IP) | 0.3536 | 0.0020 | 0.0452 |
| SELENOP | selenoprotein P(SELENOP) | 1.4299 | 0.0000 | 0.0000 |
| SEMA3B | semaphorin 3B(SEMA3B) | -1.5900 | 0.0000 | 0.0015 |
| SEMA5A | semaphorin 5A(SEMA5A) | -0.8943 | 0.0004 | 0.0142 |
| SEPHS2 | selenophosphate synthetase 2(SEPHS2) | 0.3465 | 0.0011 | 0.0292 |
| SEPTIN9 | Septin 9 | -0.6340 | 0.0003 | 0.0103 |
| SERAC1 | serine active site containing 1(SERAC1) | 0.8519 | 0.0001 | 0.0042 |
| SERINC3 | serine incorporator 3(SERINC3) | -0.5252 | 0.0016 | 0.0384 |
| SESN2 | sestrin 2(SESN2) | 0.6681 | 0.0000 | 0.0001 |
| SFRP4 | secreted frizzled related protein 4(SFRP4) | -3.0215 | 0.0005 | 0.0171 |
| SGMS2 | sphingomyelin synthase 2(SGMS2) | -1.0588 | 0.0001 | 0.0034 |
| SH2D5 | SH2 domain containing 5(SH2D5) | -1.0802 | 0.0001 | 0.0039 |
| SH3BGRL3 | SH3 domain binding glutamate rich protein like 3(SH3BGRL3) | -0.5811 | 0.0002 | 0.0074 |
| SH3RF1 | SH3 domain containing ring finger 1(SH3RF1) | -0.8657 | 0.0000 | 0.0000 |
| SHISA4 | shisa family member 4(SHISA4) | -0.4384 | 0.0001 | 0.0031 |
| SHMT2 | serine hydroxymethyltransferase 2(SHMT2) | 0.4713 | 0.0000 | 0.0010 |
| SHPRH | SNF2 histone linker PHD RING helicase (SHPRH) | 0.6954 | 0.0014 | 0.0343 |
| SHROOM2 | shroom family member 2(SHROOM2) | -3.0339 | 0.0003 | 0.0109 |
| SIAH2 | siah E3 ubiquitin protein ligase 2(SIAH2) | 0.4481 | 0.0001 | 0.0043 |
| SIGMAR1 | sigma non-opioid intracellular receptor 1(SIGMAR1) | -0.3140 | 0.0016 | 0.0385 |
| SLC13A4 | solute carrier family 13 member 4(SLC13A4) | -1.3055 | 0.0016 | 0.0379 |
| SLC16A4 | solute carrier family 16 member 4(SLC16A4) | -0.9449 | 0.0016 | 0.0375 |
| SLC18B1 | solute carrier family 18 member B1(SLC18B1) | 1.2289 | 0.0004 | 0.0140 |
| SLC1A3 | solute carrier family 1 member 3(SLC1A3) | 0.6073 | 0.0001 | 0.0042 |
| SLC20A2 | solute carrier family 20 member 2(SLC20A2) | -1.0891 | 0.0000 | 0.0000 |
| SLC2A11 | solute carrier family 2 member 11(SLC2A11) | -0.8135 | 0.0000 | 0.0002 |
| SLC35E2A | Solute Carrier Family 35 Member E2A | -0.8760 | 0.0011 | 0.0279 |
| SLC35E4 | solute carrier family 35 member E4(SLC35E4) | -1.0919 | 0.0000 | 0.0008 |
| SLC39A8 | solute carrier family 39 member 8(SLC39A8) | 1.5875 | 0.0019 | 0.0423 |
| SLC40A1 | solute carrier family 40 member 1(SLC40A1) | 1.7366 | 0.0019 | 0.0428 |
| SLC4A11 | solute carrier family 4 member 11(SLC4A11) | -1.3263 | 0.0000 | 0.0003 |
| SLC4A4 | solute carrier family 4 member 4(SLC4A4) | -1.0552 | 0.0001 | 0.0061 |
| SLC6A9 | solute carrier family 6 member 9(SLC6A9) | 0.8106 | 0.0003 | 0.0100 |
| SLC9A7 | solute carrier family 9 member A7(SLC9A7) | -1.0647 | 0.0000 | 0.0019 |
| SMG1P3 | SMG1P3, nonsense mediated mRNA decay associated PI3K related kinase pseudogene 3(SMG1P3) | -0.9728 | 0.0001 | 0.0061 |
| SMPD1 | sphingomyelin phosphodiesterase 1(SMPD1) | -1.1262 | 0.0001 | 0.0063 |
| SMURF2 | SMAD specific E3 ubiquitin protein ligase 2(SMURF2) | -0.7436 | 0.0000 | 0.0014 |
| SNORA73A | small nucleolar RNA, H/ACA box 73A(SNORA73A) | -4.9739 | 0.0006 | 0.0178 |
| SNX21 | sorting nexin family member 21(SNX21) | -0.5896 | 0.0001 | 0.0042 |
| SORT1 | sortilin 1(SORT1) | -1.1269 | 0.0001 | 0.0035 |
| SOX4 | SRY-box 4(SOX4) | 1.1661 | 0.0000 | 0.0000 |
| SPACA6 | sperm acrosome associated 6(SPACA6) | -0.8253 | 0.0001 | 0.0042 |
| SPART | Spartin | 0.4609 | 0.0001 | 0.0049 |
| SPON1 | spondin 1(SPON1) | 6.1499 | 0.0000 | 0.0000 |
| SPTLC2 | serine palmitoyltransferase long chain base subunit 2(SPTLC2) | -0.6049 | 0.0019 | 0.0438 |
| SQOR | Sulfide Quinone Oxidoreductase | -0.7724 | 0.0007 | 0.0209 |
| SRCAP | Snf2 related CREBBP activator protein (SRCAP) | 0.4165 | 0.0023 | 0.0488 |
| SRGAP2B | SLIT-ROBO Rho GTPase activating protein 2B(SRGAP2B) | 0.4822 | 0.0013 | 0.0334 |
| SRGAP2D | SLIT-ROBO Rho GTPase activating protein 2D (pseudogene)(SRGAP2D) | 1.7082 | 0.0022 | 0.0467 |
| SRP54 | signal recognition particle 54(SRP54) | 0.3824 | 0.0001 | 0.0056 |
| ST6GALNAC5 | ST6 N-acetylgalactosaminide alpha-2,6-sialyltransferase 5(ST6GALNAC5) | -0.9918 | 0.0011 | 0.0288 |
| ST6GALNAC6 | ST6 N-acetylgalactosaminide alpha-2,6-sialyltransferase 6(ST6GALNAC6) | -0.4336 | 0.0008 | 0.0224 |
| STARD5 | StAR related lipid transfer domain containing 5(STARD5) | -0.8347 | 0.0006 | 0.0176 |
| STING1 | Stimulator Of Interferon Response CGAMP Interactor 1 | -0.5099 | 0.0000 | 0.0001 |
| STK38L | serine/threonine kinase 38 like (STK38L) | 0.6627 | 0.0003 | 0.0106 |
| STMN2 | stathmin 2(STMN2) | 5.0619 | 0.0000 | 0.0015 |
| STMN3 | stathmin 3(STMN3) | -1.3951 | 0.0003 | 0.0097 |
| STS | steroid sulfatase (microsomal), isozyme S(STS) | -0.9721 | 0.0000 | 0.0000 |
| SYBU | syntabulin (SYBU) | 2.7922 | 0.0000 | 0.0000 |
| SYNE1 | spectrin repeat containing nuclear envelope protein 1(SYNE1) | 0.8581 | 0.0002 | 0.0067 |
| SYNGR2 | synaptogyrin 2(SYNGR2) | -1.3205 | 0.0000 | 0.0001 |
| TACC2 | transforming acidic coiled-coil containing protein 2(TACC2) | -0.8993 | 0.0000 | 0.0002 |
| TAFA5 | TAFA Chemokine Like Family Member 5 | -1.2035 | 0.0000 | 0.0017 |
| TANGO2 | transport and golgi organization 2 homolog (TANGO2) | -0.4127 | 0.0006 | 0.0192 |
| TAPBP | TAP binding protein (TAPBP) | -0.8226 | 0.0005 | 0.0157 |
| TARS1 | Threonyl-TRNA Synthetase 1 | 0.4725 | 0.0000 | 0.0007 |
| TBC1D2 | TBC1 domain family member 2(TBC1D2) | -1.0153 | 0.0000 | 0.0000 |
| TBC1D3E | TBC1 domain family member 3E(TBC1D3E) | -21.7207 | 0.0000 | 0.0000 |
| TBC1D3G | TBC1 domain family member 3G(TBC1D3G) | -22.0080 | 0.0000 | 0.0000 |
| TBC1D3K | TBC1 domain family member 3K(TBC1D3K) | -21.9529 | 0.0000 | 0.0000 |
| TBX2 | T-box 2(TBX2) | -1.2766 | 0.0000 | 0.0012 |
| TCEA1 | transcription elongation factor A1(TCEA1) | 0.6348 | 0.0000 | 0.0000 |
| TCF7L2 | transcription factor 7 like 2(TCF7L2) | 0.6342 | 0.0006 | 0.0190 |
| TCIM | Transcriptional And Immune Response Regulator | 1.5278 | 0.0000 | 0.0004 |
| TCP1 | t-complex 1(TCP1) | 0.2996 | 0.0016 | 0.0375 |
| TCTA | T-cell leukemia translocation altered (TCTA) | -0.3387 | 0.0016 | 0.0385 |
| TDRD9 | tudor domain containing 9(TDRD9) | 6.7596 | 0.0000 | 0.0027 |
| TECR | trans-2,3-enoyl-CoA reductase (TECR) | -0.4494 | 0.0023 | 0.0494 |
| TEDC1 | Tubulin Epsilon and Delta Complex 1 | -1.4449 | 0.0000 | 0.0000 |
| TFPI | tissue factor pathway inhibitor (TFPI) | -0.9982 | 0.0000 | 0.0026 |
| TGFB3 | transforming growth factor beta 3(TGFB3) | 0.7907 | 0.0001 | 0.0041 |
| TGFBR3 | transforming growth factor beta receptor 3(TGFBR3) | -1.2648 | 0.0002 | 0.0077 |
| THSD1 | thrombospondin type 1 domain containing 1(THSD1) | -1.7600 | 0.0000 | 0.0013 |
| THY1 | Thy-1 cell surface antigen (THY1) | -0.6983 | 0.0006 | 0.0192 |
| TJP2 | tight junction protein 2(TJP2) | -1.1266 | 0.0000 | 0.0006 |
| TKT | transketolase (TKT) | -0.4637 | 0.0018 | 0.0411 |
| TLE1 | transducin like enhancer of split 1(TLE1) | -0.8108 | 0.0000 | 0.0000 |
| TLX2 | T-cell leukemia homeobox 2(TLX2) | -3.9318 | 0.0001 | 0.0052 |
| TMBIM1 | transmembrane BAX inhibitor motif containing 1(TMBIM1) | -0.8040 | 0.0000 | 0.0007 |
| TMEM106C | transmembrane protein 106C(TMEM106C) | -0.4372 | 0.0002 | 0.0088 |
| TMEM120A | transmembrane protein 120A(TMEM120A) | -0.4866 | 0.0007 | 0.0208 |
| TMEM131L | Transmembrane 131 Like | -0.8242 | 0.0002 | 0.0085 |
| TMEM132D | transmembrane protein 132D(TMEM132D) | -7.6383 | 0.0005 | 0.0157 |
| TMEM155 | transmembrane protein 155(TMEM155) | 1.8723 | 0.0000 | 0.0000 |
| TMEM176B | transmembrane protein 176B(TMEM176B) | 3.7462 | 0.0006 | 0.0181 |
| TMEM268 | transmembrane protein 268(TMEM268) | 0.5352 | 0.0004 | 0.0135 |
| TMEM50A | transmembrane protein 50A(TMEM50A) | -0.4837 | 0.0016 | 0.0387 |
| TMEM53 | transmembrane protein 53(TMEM53) | -0.5830 | 0.0016 | 0.0384 |
| TMEM8B | transmembrane protein 8B(TMEM8B) | -0.5112 | 0.0007 | 0.0210 |
| TMTC2 | transmembrane and tetratricopeptide repeat containing 2(TMTC2) | 1.2282 | 0.0000 | 0.0000 |
| TNC | tenascin C(TNC) | 2.5881 | 0.0000 | 0.0000 |
| TNFAIP3 | TNF alpha induced protein 3(TNFAIP3) | -1.3651 | 0.0001 | 0.0035 |
| TNFAIP6 | TNF alpha induced protein 6(TNFAIP6) | 1.9491 | 0.0000 | 0.0000 |
| TNFRSF10C | TNF receptor superfamily member 10c(TNFRSF10C) | -2.3742 | 0.0017 | 0.0393 |
| TNFRSF1B | TNF receptor superfamily member 1B(TNFRSF1B) | -1.7352 | 0.0006 | 0.0189 |
| TNFRSF25 | TNF receptor superfamily member 25(TNFRSF25) | -0.8975 | 0.0021 | 0.0466 |
| TNIP1 | TNFAIP3 interacting protein 1(TNIP1) | -0.3600 | 0.0011 | 0.0283 |
| TPRG1L | tumor protein p63 regulated 1 like (TPRG1L) | -0.3398 | 0.0002 | 0.0081 |
| TRAK1 | trafficking kinesin protein 1(TRAK1) | -0.4590 | 0.0006 | 0.0189 |
| TRAK2 | trafficking kinesin protein 2(TRAK2) | -0.5974 | 0.0000 | 0.0001 |
| TRH | thyrotropin releasing hormone (TRH) | -3.8444 | 0.0001 | 0.0063 |
| TRIB3 | tribbles pseudokinase 3(TRIB3) | 1.3727 | 0.0000 | 0.0000 |
| TRIL | TLR4 interactor with leucine rich repeats (TRIL) | 3.4277 | 0.0000 | 0.0001 |
| TRIM32 | tripartite motif containing 32(TRIM32) | 0.4071 | 0.0016 | 0.0383 |
| TRIM47 | tripartite motif containing 47(TRIM47) | -1.0113 | 0.0007 | 0.0215 |
| TRPS1 | transcriptional repressor GATA binding 1(TRPS1) | 0.5034 | 0.0000 | 0.0024 |
| TSFM | Ts translation elongation factor, mitochondrial (TSFM) | 0.4978 | 0.0013 | 0.0318 |
| TSHR | thyroid stimulating hormone receptor (TSHR) | 4.4429 | 0.0003 | 0.0114 |
| TSPAN18 | tetraspanin 18(TSPAN18) | 2.6062 | 0.0000 | 0.0000 |
| TSPAN2 | tetraspanin 2(TSPAN2) | 1.8745 | 0.0007 | 0.0216 |
| TSPAN4 | tetraspanin 4(TSPAN4) | -0.7222 | 0.0021 | 0.0464 |
| TTC39B | tetratricopeptide repeat domain 39B(TTC39B) | 0.8691 | 0.0000 | 0.0001 |
| TTI2 | TELO2 interacting protein 2(TTI2) | 0.5784 | 0.0011 | 0.0281 |
| TTLL3 | tubulin tyrosine ligase like 3(TTLL3) | -0.3926 | 0.0023 | 0.0490 |
| TUBE1 | tubulin epsilon 1(TUBE1) | 0.7518 | 0.0000 | 0.0000 |
| TYRO3 | TYRO3 protein tyrosine kinase (TYRO3) | -0.5601 | 0.0007 | 0.0204 |
| UBE2D4 | ubiquitin conjugating enzyme E2 D4 (putative)(UBE2D4) | -0.4547 | 0.0015 | 0.0358 |
| UBE2J1 | ubiquitin conjugating enzyme E2 J1(UBE2J1) | 0.2902 | 0.0012 | 0.0315 |
| UGP2 | UDP-glucose pyrophosphorylase 2(UGP2) | 0.5577 | 0.0000 | 0.0027 |
| UHRF1BP1 | UHRF1 binding protein 1(UHRF1BP1) | 0.6790 | 0.0021 | 0.0466 |
| ULBP3 | UL16 binding protein 3(ULBP3) | -0.8690 | 0.0000 | 0.0006 |
| UQCC1 | ubiquinol-cytochrome c reductase complex assembly factor 1(UQCC1) | -0.5303 | 0.0000 | 0.0019 |
| USP32P1 | ubiquitin specific peptidase 32 pseudogene 1(USP32P1) | 5.1588 | 0.0000 | 0.0000 |
| USP32P3 | ubiquitin specific peptidase 32 pseudogene 3(USP32P3) | 2.4374 | 0.0000 | 0.0007 |
| VAMP1 | vesicle associated membrane protein 1(VAMP1) | -1.0559 | 0.0014 | 0.0336 |
| VAMP5 | vesicle associated membrane protein 5(VAMP5) | -0.5569 | 0.0006 | 0.0176 |
| VDR | vitamin D (1,25- dihydroxyvitamin D3) receptor (VDR) | 0.7871 | 0.0005 | 0.0171 |
| VEGFB | vascular endothelial growth factor B(VEGFB) | -0.6381 | 0.0000 | 0.0000 |
| VHL | von Hippel-Lindau tumor suppressor (VHL) | 0.4844 | 0.0000 | 0.0026 |
| VIT | vitrin (VIT) | -2.1439 | 0.0000 | 0.0006 |
| VPS9D1 | VPS9 domain containing 1(VPS9D1) | -0.4514 | 0.0019 | 0.0431 |
| WARS1 | Tryptophanyl-TRNA Synthetase 1 | 0.7520 | 0.0000 | 0.0000 |
| WNT5B | Wnt family member 5B(WNT5B) | -0.7916 | 0.0005 | 0.0151 |
| WNT9A | Wnt family member 9A(WNT9A) | -0.9739 | 0.0015 | 0.0360 |
| XG | Xg blood group (XG) | -1.1400 | 0.0000 | 0.0005 |
| XKR8 | XK related 8(XKR8) | -0.5845 | 0.0004 | 0.0133 |
| XPOT | exportin for tRNA (XPOT) | 0.5938 | 0.0000 | 0.0000 |
| YARS1 | Tyrosyl-TRNA Synthetase 1 | 0.3518 | 0.0010 | 0.0270 |
| ZBTB47 | zinc finger and BTB domain containing 47(ZBTB47) | -0.5276 | 0.0000 | 0.0003 |
| ZDHHC12 | zinc finger DHHC-type containing 12(ZDHHC12) | -0.6469 | 0.0001 | 0.0039 |
| ZDHHC3 | zinc finger DHHC-type containing 3(ZDHHC3) | -0.5707 | 0.0000 | 0.0014 |
| ZFAND5 | zinc finger AN1-type containing 5(ZFAND5) | -0.3712 | 0.0016 | 0.0375 |
| ZFPM2 | zinc finger protein, FOG family member 2(ZFPM2) | 2.6636 | 0.0002 | 0.0078 |
| ZFYVE16 | zinc finger FYVE-type containing 16(ZFYVE16) | 0.6565 | 0.0000 | 0.0014 |
| ZNF22 | zinc finger protein 22(ZNF22) | 0.7277 | 0.0010 | 0.0275 |
| ZNF334 | zinc finger protein 334(ZNF334) | 2.6140 | 0.0000 | 0.0000 |
| ZNF385D | zinc finger protein 385D(ZNF385D) | -1.0770 | 0.0003 | 0.0110 |
| ZNF624 | zinc finger protein 624(ZNF624) | 1.0290 | 0.0006 | 0.0192 |
| ZNF704 | zinc finger protein 704(ZNF704) | 3.7128 | 0.0000 | 0.0000 |
| ZNF770 | zinc finger protein 770(ZNF770) | 0.5909 | 0.0002 | 0.0075 |
| ZSWIM4 | zinc finger SWIM-type containing 4(ZSWIM4) | 0.9554 | 0.0002 | 0.0083 |

**Supplementary Table 2.** Lists of differentially expressed genes (DEGs) related to inflammation (62 DEGs), autophagy (37 DEGs) and mitochondria (42 DEGs)

| Inflammation-related genes (62 DEGs) | Autophagy-related genes (37 DEGs) | Mitochondrial-related genes (42 DEGs) |
| --- | --- | --- |
| LAMA5 | FZD1 | LONP1 |
| TNFRSF1B | GAB1 | ACADM |
| NFKB2 | LMNA | HSPA9 |
| STING1 | RAF1 | UQCC1 |
| TRIM32 | EIF4EBP1 | TSFM |
| POLR2L | CCND1 | ALDH4A1 |
| ITPR3 | IL20RB | ECHDC2 |
| RAF1 | RAB27B | ETHE1 |
| PIK3R3 | IL21R | MT-ATP6 |
| EGFR | ITGA3 | MT-CO2 |
| MAP2K3 | HSP90AA1 | GLS |
| JUN | RAB15 | SQOR |
| HTR2A | H2AC19 | ALDH1B1 |
| IGF1 | HSBP1 | CHDH |
| BDKRB1 | LAMTOR4 | SHMT2 |
| RAPGEF1 | PRKAB2 | MTHFD2 |
| CCND1 | JUN | ALDH18A1 |
| GNAI2 | MAPKAPK3 | LYPLA1 |
| TCF7L2 | VHL | ALDH1L2 |
| NOTCH3 | CITED2 | GPT2 |
| PPP3CA | NOX4 | MT-CO1 |
| HIF1A | CBX4 | MT-ND2 |
| HSP90AA1 | GPX3 | MT-ND5 |
| NFATC2 | HMGA1 | PCK2 |
| IL21R | NUP98 | CHPT1 |
| BCL2L1 | MAP2K3 | GARS1 |
| TGFB3 | ETS2 | MT-ND3 |
| *CD44* | HIF1A | ARG2 |
| *CD81* | HSPA9 | FAM210B |
| *CTSB* | PSMB2 | CBR3 |
| NEFL | IGF1 | LDHB |
| PDGFB | IRS2 | CYB5R3 |
| IRS2 | SESN2 | FKBP8 |
| NEDD4 | PCK2 | MT-ND1 |
| PRL | CAMKK1 | GPAM |
| PSMB2 | PIK3R3 | PRXL2A |
| NUP98 | PPP1R12A | MGARP |
| IL20RB |  | SPTLC2 |
| TNFRSF25 |  | BCL2L1 |
| RASA2 |  | GPAT2 |
| HLA-B |  | DMPK |
| IL17RB |  | SERAC1 |
| HLA-C |  |  |
| HLA-E |  |  |
| CTSC |  |  |
| TRIB3 |  |  |
| UBE2J1 |  |  |
| PJA1 |  |  |
| BTN3A2 |  |  |
| UBE2D4 |  |  |
| GAB1 |  |  |
| CLEC2B |  |  |
| SIAH2 |  |  |
| SMURF2 |  |  |
| ICAM5 |  |  |
| ENAH |  |  |
| ULBP3 |  |  |
| VHL |  |  |
| TAPBP |  |  |
| KIF3B |  |  |
| FBXW5 |  |  |
| SH3RF1 |  |  |

**Supplementary Table 3.** Concentration of secreted inflammatory cytokines in IBM vs CTL supernatants. IBM fibroblasts revealed an increased expression of most of these cytokines.

| **CYTOKINE** | **CYTOKINE NAME** | **CTL (n=12)** | **IBM (n=13)** | **IBM/CTL** | **SEM IBM/MEAN CTL** |
| --- | --- | --- | --- | --- | --- |
| **GCSF** | Granulocyte colony-stimulating factor | 11.01 ± 9.45 | 146.42 ± 118.15 | 13.30 | 1,62 |
| **TNFα** | Tumor necrosis factor α | 0.33 ± 0.33 | 3.34 ± 1.51 | 10.12 | 0,40 |
| **GMCSF** | Granulocyte-macrophage colony-stimulating factor | 0.12 ± 0.12 | 0.62 ± 0.40 | 5.17 | 10,73 |
| **eotaxin*** | C-C motif chemokine 11 (CCL11) | 4.81 ± 4.10 | 20.18 ± 7.80 | 4.20 | 3,33 |
| **IL7** | interleukin 7 | 0.55 ± 0.55 | 2.12 ± 1.04 | 3.85 | 0,44 |
| **MIP1a** | C-C motif chemokine 3 (CCL3) | 0.05 ± 0.03 | 0.15 ± 0.08 | 3.00 | 0,78 |
| **IL4** | interleukin 4 | 0.31 ± 0.24 | 0.83 ± 0.25 | 2.68 | 0,46 |
| **IL8** | interleukin 8 | 829.27 ± 325.46 | 2106.95 ± 746.50 | 2.54 | 0,81 |
| **IL13** | interleukin 13 | 0.08 ± 0.06 | 0.20 ± 0.07 | 2.50 | 0,29 |
| **MCP1** | Monocyte Chemoattractant Protein-1 | 162.62 ± 38.64 | 394.38 ± 100.04 | 2.43 | 1,89 |
| **RANTES** | C-C motif chemokine 5 (CCL5) | 5.14 ± 3.70 | 11.24 ± 6.98 | 2.19 | 0,90 |
| **IL17** | interleukin 17 | 0.27 ± 0.27 | 0.55 ± 0.29 | 2.04 | 0,46 |
| **IL1β** | Interleukin 1β | 0.09 ± 0.09 | 0.18 ± 0.07 | 2.00 | 0,88 |
| **IL9** | interleukin 9 | 7.85 ± 3.23 | 13.14 ± 3.63 | 1.67 | 1,07 |
| **MIP1b** | C-C motif chemokine 4 (CCL4) | 2.57 ± 1.36 | 4.02 ± 1.43 | 1.56 | 0,24 |
| **FGFbasic** | Basic fibroblast growth factor | 3.14 ± 1.49 | 3.84 ± 1.26 | 1.22 | 0,62 |
| **IL1ra** | Interleukin-1 receptor antagonist protein | 6.49 ± 6.27 | 7.89 ± 2.99 | 1.22 | 1,60 |
| **IFNg** | Interferon gamma | 10.47 ± 8.04 | 11.50 ± 4.60 | 1.10 | 0,56 |
| **IL6** | interleukin 6 | 778.34 ± 330.12 | 722.18 ± 224.65 | 0.93 | 1,36 |
| **VEGF** | Vascular endothelial growth factor | 67.93 ± 21.57 | 60.36 ± 21.65 | 0.89 | 4,58 |
| **IP10** | C-X-C motif chemokine ligand 10 (CXCL10) | 63.01 ± 51.9 | 39.60 ± 15.23 | 0.63 | 0,32 |

n=13 IBM vs 12 CTL; *p-value<0.05, Mann–Whitney U test; IBM: inclusion body myositis; CTL: controls. Color code for ratio IBM/CTL: <1 in blue (for decreased expression of cytokines in IBM); =1 in white (for equal expression between cohorts); and >1 in red (for higher expression in IBM). Mean fold change ratio (and deviation) of secreted cytokines in IBM patients vs. CTL fibroblasts, normalized by cell number, displayed in Fig 2B.

**Supplementary Table 4.** Expression of 20 autophagy proteins in IBM vs. CTL fibroblasts. Most of these proteins displayed a decreased expression in IBM, suggesting a reduced activity of the autophagy process.

| **Autophagy proteins** | **Autophagy protein name** | **CTL (n=8)** | **IBM (n=8)** | **IBM/CTL** | **SEM IBM/MEAN CTL** |
| --- | --- | --- | --- | --- | --- |
| DDR2 | Discoidin domain-containing receptor 2 | 1174.88 ± 187.39 | 1150.5 ± 215.46 | 0.98 | 0,18 |
| BNIP3L | BCL2/adenovirus E1B 19 kDa protein-interacting protein 3-like | 2386.88 ± 374.14 | 2262 ± 349.84 | 0.95 | 0,15 |
| ATG5 | Autophagy related protein 5 | 1814.5 ± 327.55 | 1688.25 ± 398.4 | 0.93 | 0,22 |
| ATG4A | Autophagy Related 4A Cysteine Peptidase | 2707.38 ± 442.75 | 2454.38 ± 398.88 | 0.91 | 0,15 |
| ATG3 | Autophagy related protein 3 | 1889.88 ± 257.07 | 1682.13 ± 278.41 | 0.89 | 0,15 |
| Rheb | Ras Homolog, MTORC1 Binding protein | 1674.75 ± 224.93 | 1481.88 ± 301.1 | 0.88 | 0,18 |
| ATG7 | Autophagy related protein 7 | 2004.63 ± 307.41 | 1719.75 ± 291.61 | 0.86 | 0,15 |
| ATG10 | Autophagy related protein 10 | 2582.38 ± 351.96 | 2156.13 ± 314.94 | 0.83 | 0,12 |
| ATG13 | Autophagy related protein 13 | 2159.13 ± 303.39 | 1801.38 ± 204.21 | 0.83 | 0,09 |
| Beclin | Beclin 1 | 1466.75 ± 243.15 | 1222.63 ± 170.38 | 0.83 | 0,12 |
| ATG4B | Autophagy Related 4B Cysteine Peptidase | 1411.88 ± 297.45 | 1149.5 ± 243.53 | 0.81 | 0,17 |
| P62 | Sequestosome 1 (SQSTM1) | 17700.13 ± 2737.31 | 14212.38 ± 2118.71 | 0.8 | 0,12 |
| LC3B | Microtubule-associated proteins 1A/1B light chain 3B | 6540.13 ± 1259.3 | 5162.88 ± 679.67 | 0.79 | 0,10 |
| GABARAP | Gamma-aminobutyric acid receptor-associated protein | 1574.38 ± 199.19 | 1244.13 ± 165.29 | 0.79 | 0,10 |
| LAMP1 | Lysosome-associated membrane glycoprotein 1 | 2147 ± 320.11 | 1645.63 ± 208.2 | 0.77 | 0,10 |
| Alpha-Synuclein | Alpha-Synuclein (SNCA gene) | 1503.75 ± 144.86 | 1146.88 ± 149.84 | 0.76 | 0,10 |
| LC3A | Microtubule-associated proteins 1A/1B light chain 3A | 5914 ± 928.3 | 4432.13 ± 640.62 | 0.75 | 0,11 |
| NBS1 | Nibrin | 5491.88 ± 1202 | 3654 ± 334.12 | 0.67 | 0,06 |
| ATG12 | Autophagy related protein 12 | 4844.13 ± 1173.43 | 3139 ± 399.84 | 0.65 | 0,08 |
| MSK1 | Ribosomal Protein S6 Kinase A5 | 485.13 ± 115.21 | 311.75 ± 44.59 | 0.64 | 0,09 |

Abbreviations: IBM: Inclusion Body Myositis; CTL: healthy control subjects. Concentration of each protein in the autophagy array presented as mean ± SEM or ratio IBM/CTL per each autophagic protein (n=8/group). Color code for ratio IBM/CTL: <1 in blue (for decreased expression of cytokines in IBM) and =1 in white (for equal expression between cohorts). Mean fold change ratio (and deviation) of autophagy proteins in IBM patients vs. CTL fibroblasts, normalized by protein content, displayed in Fig 3B.

**Supplementary Table 5**. Differentially expressed genes (DEGs) related to metabolite concentration in fibroblasts. The table depicts the relationship of 10 DEGs involved in metabolite metabolism with their respective alteration at amino acids and organic acids level, to relate expression vs. metabolism patterns in IBM vs. CTL fibroblasts.

| Gene name | Description | Expression pattern | Metabolite pattern | Pathway |
| --- | --- | --- | --- | --- |
| PSAT1 | phosphoserine aminotransferase 1 | upregulated | Increased serine and glycine | vitamin B6 pathway |
| ETHE1 | ETHE1 persulfate dioxygenase | downregulated | Increase lactate, ethylmalonic and methyl succinic | Mitochondrial metabolism |
| MTND5 | NADH dehydrogenase, subunit 5 (complex I) | downregulated | Increased lactate, alanine and TCA cycle | Mitochondrial metabolism |
| ARG2 | arginase 2 | upregulated | Reduced arginine | Mitochondrial metabolism |
| MTCO2 | cytochrome c oxidase subunit II | downregulated | Increased lactate, alanine and TCA cycle | Mitochondrial metabolism |
| MTATP6 | ATP synthase F0 subunit 6 | downregulated | Increased lactate, alanine and TCA cycle | Mitochondrial metabolism |
| GPX3 | glutathione peroxidase isoform 3 | upregulated | Could be related to high pyroglutamic acid | glutathione metabolism |
| SQOR | sulphide quinone oxidoreductase | downregulated | Related to ETHE1 | Mitochondrial metabolism |
| GPT2 | glutamic--pyruvic transaminase 2 | upregulated | Increased glutamate and alanine | Mitochondrial metabolism |
| SERAC1 | serine active site containing 1 | upregulated | Increased serine | Mitochondrial metabolism |

Abbreviations: TCA: Tricarboxylic Acid Cycle.

**Supplementary Table 6**. Organic acids in IBM vs. CTL fibroblasts. Organic acids showed a general increase in IBM, suggesting a deregulation of intermediary metabolism related to mitochondrial function, as many organic acids are involved in tricarboxylic acid cycle (TCA) that further feeds the mitochondrial respiratory chain.

| **ORGANIC ACIDS**  **(nmol/mg protein)** | **CTL (n=10)** | **IBM (n=11)** | **IBM/CTL** | **SEM IBM/MEAN CTL** |
| --- | --- | --- | --- | --- |
| **2OHglutaric** | **0.33 ± 0.1** | **1.48 ± 0.62** | 4.48 | 1,88 |
| 2-hidroxivaleric | 0.3 ± 0.15 | 1.08 ± 0.38 | 3.60 | 1,27 |
| a-ketoglutarate | 3.9 ± 1.48 | 9.28 ± 3.86 | 2.38 | 0,99 |
| **succinic** | **0.07 ± 0.01** | **0.16 ± 0.06** | 2.29 | 0,86 |
| **lactic** | **0.26 ± 0.05** | **0.51 ± 0.06** | 1.96 | 0,23 |
| citric | 178.23 ± 56.65 | 322.66 ± 150.86 | 1.81 | 0,85 |
| fumaric | 0.68 ± 0.17 | 1.17 ± 0.18 | 1.72 | 0,26 |
| **sebacic** | **0.11 ± 0.01** | **0.18 ± 0.01** | 1.64 | 0,09 |
| **pyroglutamic** | **32.04 ± 2.6** | **44.71 ± 2.58** | 1.40 | 0,08 |
| malic | 0.9 ± 0.17 | 1.15 ± 0.2 | 1.28 | 0,22 |
| glutaric | 0.2 ± 0.02 | 0.25 ± 0.05 | 1.25 | 0,25 |
| adipic | 0.41 ± 0.03 | 0.48 ± 0.04 | 1.17 | 0,10 |
| suberic | 0.72 ± 0.07 | 0.79 ± 0.05 | 1.10 | 0,07 |
| ethylmalonic | 0.26 ± 0.01 | 0.26 ± 0.02 | 1.00 | 0,08 |
| glycerol | 5.09 ± 0.37 | 5.05 ± 0.61 | 0.99 | 0,12 |
| 3/OH/Sebacic | 0.15 ± 0.03 | 0.14 ± 0.02 | 0.93 | 0,13 |
| **oxalate** | **36.33 ± 0.83** | **32.34 ± 2.03** | 0.89 | 0,06 |

Abbreviations: IBM: inclusion body myositis; CTL: healthy control subjects. N=11 IBM vs. 10 CTL; ***p-value<0.05**, Mann–Whitney U test. Color code for ratio IBM/CTL: <1 in blue (for decreased expression of metabolites in IBM); =1 in white (for equal expression between cohorts); and >1 in red (for higher expression in IBM). Concentration of each analyte presented as mean ± SEM and normalized by protein content. Mean fold change ratio (and deviation) of organic acids in IBM patients vs. CTL fibroblasts, normalized by protein content, displayed in Fig 6A.

**Supplementary Table 7**. Amino acids levels in fibroblasts of IBM patients vs. CTL fibroblasts.

| **Amino Acids (µmol/g)** | **CTL (n=10)** | **IBM (n=11)** | **IBM/CTL** | **SEM IBM/MEAN CTL** |
| --- | --- | --- | --- | --- |
| Ala | 2.26 ± 0.16 | 2.63 ± 0.09 | 1.16 | 0,04 |
| Asn | 0.62 ± 0.04 | 0.72 ± 0.04 | 1.16 | 0,06 |
| Ser | 3.16 ± 0.13 | 3.52 ± 0.12 | 1.11 | 0,04 |
| Arg | 1.39 ± 0.1 | 1.52 ± 0.05 | 1.09 | 0,04 |
| Tyr | 1.21 ± 0.02 | 1.26 ± 0.02 | 1.04 | 0,02 |
| Val | 1.8 ± 0.04 | 1.84 ± 0.04 | 1.02 | 0,02 |
| Lys | 1.61 ± 0.05 | 1.63 ± 0.04 | 1.01 | 0,02 |
| Trp | 0.24 ± 0 | 0.24 ± 0.01 | 1.00 | 0,04 |
| Leu | 1.96 ± 0.04 | 1.95 ± 0.03 | 0.99 | 0,02 |
| Ile | 1.48 ± 0.04 | 1.47 ± 0.03 | 0.99 | 0,02 |
| Gly | 6.98 ± 0.36 | 6.88 ± 0.58 | 0.99 | 0,08 |
| Pro | 2.54 ± 0.22 | 2.46 ± 0.35 | 0.97 | 0,14 |
| Met | 0.58 ± 0.01 | 0.56 ± 0.01 | 0.97 | 0,02 |
| Glu | 21.51 ± 1.33 | 20.6 ± 1.54 | 0.96 | 0,07 |
| Thr | 3.46 ± 0.15 | 3.28 ± 0.11 | 0.95 | 0,03 |
| Asp | 3.47 ± 0.41 | 3.14 ± 0.41 | 0.90 | 0,12 |
| Gln | 3.81 ± 0.47 | 3.04 ± 0.43 | 0.80 | 0,11 |
| Tau | 2.91 ± 0.3 | 2.16 ± 0.42 | 0.74 | 0,14 |

Abbreviations: IBM: inclusion body myositis; CTL: healthy control subjects. N=11 IBM vs. 10 CTL. Color code for ratio IBM/CTL: <1 in blue (for decreased expression of metabolites in IBM); =1 in white (for equal expression between cohorts); and >1 in red (for higher expression in IBM). Concentration of each analyte presented as mean ± SEM and normalized by protein content and phenylalanine concentration. Mean fold change ratio (and deviation) of amino acids in IBM patients vs. CTL fibroblasts, normalized by protein content, displayed in Fig 6B.

**Supplementary Table 8.** Comparison of inflammatory, degenerative, and mitochondrial IBM hallmarks considering the evolution of IBM patients: stable vs progressive prognosis, compared to CTL fibroblasts.

| **TEST** | **CTL** | **IBM STB/CTL** | **IBM PROG/CTL** | **N (CTL-IBM STB-IBM PROG)** |
| --- | --- | --- | --- | --- |
| Secreted inflammatory cytokines | 1.00 | 5.21 | 1.75 | 12-5-8 |
| Autophagy proteins in basal conditions | 1.00 | 0.91 | 0.72 | 8-4-4 |
| Autophagy WB LC3BII | 1.00 | 0.53 | 0.52 | 11-3-8 |
| Autophagy WB p62 | 1.00 | 0.85 | 0.75 | 11-3-8 |
| Mitochondrial respiration | 1.00 | 0.85 | 0.83 | 10-3-7 |
| COX/CS | 1.00 | 0.60 | 0.52 | 10-3-7 |
| Oxidative stress (by lipid peroxidation) | 1.00 | 0.88 | 1.22 | 8-2-7 |
| Total Antioxidant Capacity | 1.00 | 1.68 | 2.73 | 4-4-7 |
| Mitochondrial membrane potential | 1.00 | 0.88 | 0.89 | 4-2-2 |
| Organic acids | 1.00 | 2.01 | 1.34 | 10-4-7 |
| Amino acids | 1.00 | 0.96 | 1.01 | 10-4-7 |

IBM: inclusion body myositis, separated in 2 groups: stable vs progressive prognosis; CTL: controls. Ratio of each parameter compared to CTL (ratio=1). Color code for ratio IBM/CTL: <1 in blue (for decreased expression in IBM); =1 in white (for equal expression in IBM and CTL); and >1 in red (for higher expression in IBM). The number of samples varies in each test, represented in N column.
